# Comparative proteomic analysis indicates differential responses to fumonisin B_1_ (FB_1_) and hydrolysed fumonisin B_1_ (HFB_1_) in IPEC-J2 porcine epithelial cells in vitro

**DOI:** 10.1007/s12550-025-00607-z

**Published:** 2025-10-09

**Authors:** Nabeela Gamiet, Nashia Deepnarain, Stefan Abel, Hester-Mari Burger, Elisabeth Mayer, Mariska Lilly

**Affiliations:** 1 Applied Microbial and Health Biotechnology Institute (AMHBI), Cape Peninsula University of Technology, Bellville, 7535 South Africa; 2 Department of Biomedical Science, Faculty of Health and Wellness Science, Cape Peninsula University of Technology, Bellville, 7535 South Africa; 3 Unit of Research Integrity, Directorate Research Development, Office of the Deputy Vice-Chancellor: Research, Technology Innovation and Partnership, Cape Peninsula University of Technology, Bellville, 7535 South Africa; 4 Dsm-Firmenich, Animal Nutrition and Health R&D Center, Tulln, Austria

**Keywords:** Fumonisin B_1_, Hydrolysed fumonisin B_1_, Porcine intestinal epithelial cells, Proteomics

## Abstract

The intestinal epithelium is frequently exposed to environmental contaminants such as fumonisins, mycotoxins implicated in the development of mycotoxicosis across various mammalian species, with fumonisin B_1_ (FB_1_) being the most prevalent and toxic congener. Fumonisin B_1_ (FB_1_) can be enzymatically hydrolysed to produce hydrolysed fumonisin B_1_ (HFB_1_) that displays reduced inhibitory activity toward ceramide synthase. Given the central role of ceramide synthase in sphingolipid metabolism and cellular homeostasis, the reduced inhibitory activity of HFB_1_ is considered toxicologically favourable, as it is less likely to disrupt membrane integrity and critical signalling pathways. However, the toxicity of HFB_1_ remains variable across different in vitro and in vivo models. In this study, we evaluated the impact of FB_1_ and HFB_1_ on cell viability, apoptosis, and proliferation in the porcine intestinal cell line (IPEC-J2), including inflammatory responses through interleukin 8 (IL-8). Molecular mechanisms and pathways influenced by FB_1_ and HFB_1_ exposure were investigated through proteomic and bioinformatic analyses. Differentially abundant proteins (DAPs) were identified and functionally characterised using Gene Ontology analysis based on the *Sus scrofa* (domestic pig) database, revealing 52 significant DAPs between FB_1_ and HFB_1_ treatments compared to the control. Fibronectin 1 (FN1), an adhesive glycoprotein of the intestine, was consistently detected as a DAP in cells exposed to FB_1_ and HFB_1_. FB_1_ upregulates FN1, while HFB_1_ downregulates it, leading to different oncogenic pathways revealed by STRING enrichment analysis. Proteomic analysis further revealed distinct DAPs following FB_1_ and HFB_1_ exposure, implicating alterations in immune modulation (e.g. differential regulation of CD276), iron homeostasis (upregulation of FTL and FTH1), epithelial integrity (downregulation of NTN4, ST14), extracellular matrix remodelling (reduced SPARC), and angiogenesis-related pathways (decreased TINAGL1, FBLN2, SDC4) suggesting early changes in cellular signalling, stress response, and structural regulation that may be relevant to cancer biology and warrant further investigation. These findings also demonstrate that HFB_1_ activates distinct cancer-related pathways in vitro compared to FB_1_, with in vivo studies suggesting divergent mechanisms. HFB_1_ also induces more extensive protein expression changes in IPEC-J2 cells, as reflected by the greater number of DAPs and the complexity of enriched pathways. However, further investigation is needed to determine whether these changes directly contribute to cytotoxicity or represent compensatory cellular responses.

**Supplementary Information:**

The online version contains supplementary material available at 10.1007/s12550-025-00607-z.

## Introduction

The intestinal epithelium is comprised of interlinked columnar epithelial cells, such as enterocytes, which play critical roles in the absorptive and digestive functions of the intestine, alongside maintaining its barrier and immune functions (Subramanian et al. 2020). The epithelial tissue is constantly renewed every 3–5 days through a complex interplay of cell proliferation and apoptosis (Pan et al. 2018). The intestinal barrier is vital in protecting the body from harmful external agents, such as bacterial, fungal, or viral microorganisms (MacDonald and Monteleone 2005). Pathological invasions, such as microbial infections, trigger stress responses and induce cellular apoptosis, compromising barrier integrity (Apidianakis et al. 2009). Such disruptions lead to dysregulation of immune and inflammatory signalling, resulting in impaired epithelial renewal and barrier function (Zhou et al. 2017). Hence, maintaining intestinal homeostasis is crucial for optimal gut health and function.

The gastrointestinal tract serves as the first line of defence against ingested dietary substances (Szabó et al. 2023). Intestinal epithelial cells regulate the intestinal barrier function and are constantly exposed to large quantities of contaminants and toxins such as fumonisin B_1_ (FB_1_) and hydrolysed fumonisin B_1_ (HFB_1_) with subsequent negative health effects (Bouhet and Oswald 2007). FB_1_ is a low-molecular-weight secondary metabolite produced by *Fusarium* species and is well-known for causing mycotoxicosis in several mammalian species through ceramide synthase inhibition (Bertero et al. 2018). FB_1_ exposure in pigs induces toxic effects in organs such as the lungs, liver, and kidneys (Haschek et al. 2001). Pigs are highly susceptible to FB_1_ toxicity, exhibiting nephrotoxicity, hepatotoxicity, immunotoxicity, and disruption of the intestinal barrier function (Devriendt et al. 2009; Halloy et al. 2005; Knutsen et al. 2018a, 2018b; Loiseau et al. 2007; Terciolo et al. 2019). Previous in vivo studies conducted in piglets have suggested that hydrolysed fumonisins are less toxic than fumonisins (Grenier et al. 2012). Mammalian in vivo studies have also supported this finding (Grenier et al. 2012; Hahn et al. 2015; Howard et al. 2002). For example, Flynn et al. (1997) demonstrated that HFB_1_ is approximately 100-fold less toxic than FB_1_ in cultured rat embryos. The differences in membrane permeability (LogP) and metabolic pathways between FB_1_ and HFB_1_ elucidate the variations in their toxic effects. FB_1_’s higher lipophilicity facilitates greater tissue accumulation and pronounced toxicity, while the hydrolysis to HFB_1_ significantly mitigates these effects, making HFB_1_ a less toxic compound. The potential for further metabolic conversion of HFB_1_ to more toxic derivatives underscore the complexity of its toxicological profile (Schertz et al. 2018; Antonissen et al. 2020; Neckermann et al. 2021).

Studies by Gelderblom et al. (1993) demonstrate that FB_1_ promotes liver cancer initiation in rats in vivo, whereas HFB_1_ lacks this effect. Conversely, HFB_1_ exhibits greater cytotoxicity than FB_1_ in primary hepatocytes isolated from male Fischer rats, suggesting that HFB_1_ may exert enhanced cytotoxic effects under specific in vitro conditions (Gelderblom et al. 2001). Furthermore, when HFB_1_ is exposed to palmitoyl-CoA, it becomes acylated, forming N-palmitoyl HFB_1_, which is 10 times more toxic than FB_1_ in an in vitro model (Abou-Karam et al., 2004) . At the molecular level, changes in DNA (genomics), RNA (transcriptomics), proteins (proteomics), and small metabolites (metabolomics) can be observed, providing valuable insights into the mechanisms of toxicity (Eshelli et al. 2018; González-López et al. 2021). FB_1_ can be enzymatically converted to HFB_1_, a less powerful ceramide synthase inhibitor, which has shown varying toxicity across different in vitro and in vivo models (Humpf et al. 1998; Schelstraete et al. 2020). However, the mechanisms behind these differing results remain poorly understood (Caloni et al. 2005; Dellafiora et al. 2018; Hartl and Humpf 2000; Wang et al. 2016). Studies comparing the toxicological effects of HFB_1_ and FB_1_ in mammalian models are limited, and the data thus far are inconsistent (Abbax et al. 1993; Caloni et al. 2002; Dombrink-Kurtzman 2003). To better understand the comparative cellular responses and potential cytotoxic effects, this study aimed to optimise the in vitro model by exposing porcine IPEC-J2 cells to varying concentrations of FB_1_ and HFB_1_ to assess their impact on cellular intestinal viability.

Omics studies are essential for identifying, characterising, and quantifying these biological markers to understand the mechanisms of toxicity that may harm both animals and humans (Cimbalo et al. 2022). Proteomics involves studying the collection of proteins in cells and analysing their abundance to deepen our understanding of cellular responses to toxins (Aslam et al. 2016; Piñeiro et al. 2015). By examining the different signalling pathways activated through protein analysis and pathway enrichment, we gain better insights into the mechanisms of toxicity (Kan et al. 2017). While numerous studies have investigated the effects of FB_1_ and HFB_1_ separately, few have compared their impacts at the proteome level in vitro. This study, therefore, aims to evaluate the comparative effects of FB_1_ and HFB_1_ on the porcine intestinal cell line IPEC-J2 through proteomic analysis.

## Materials and methods

### Cell culture

**Fig. 1:**
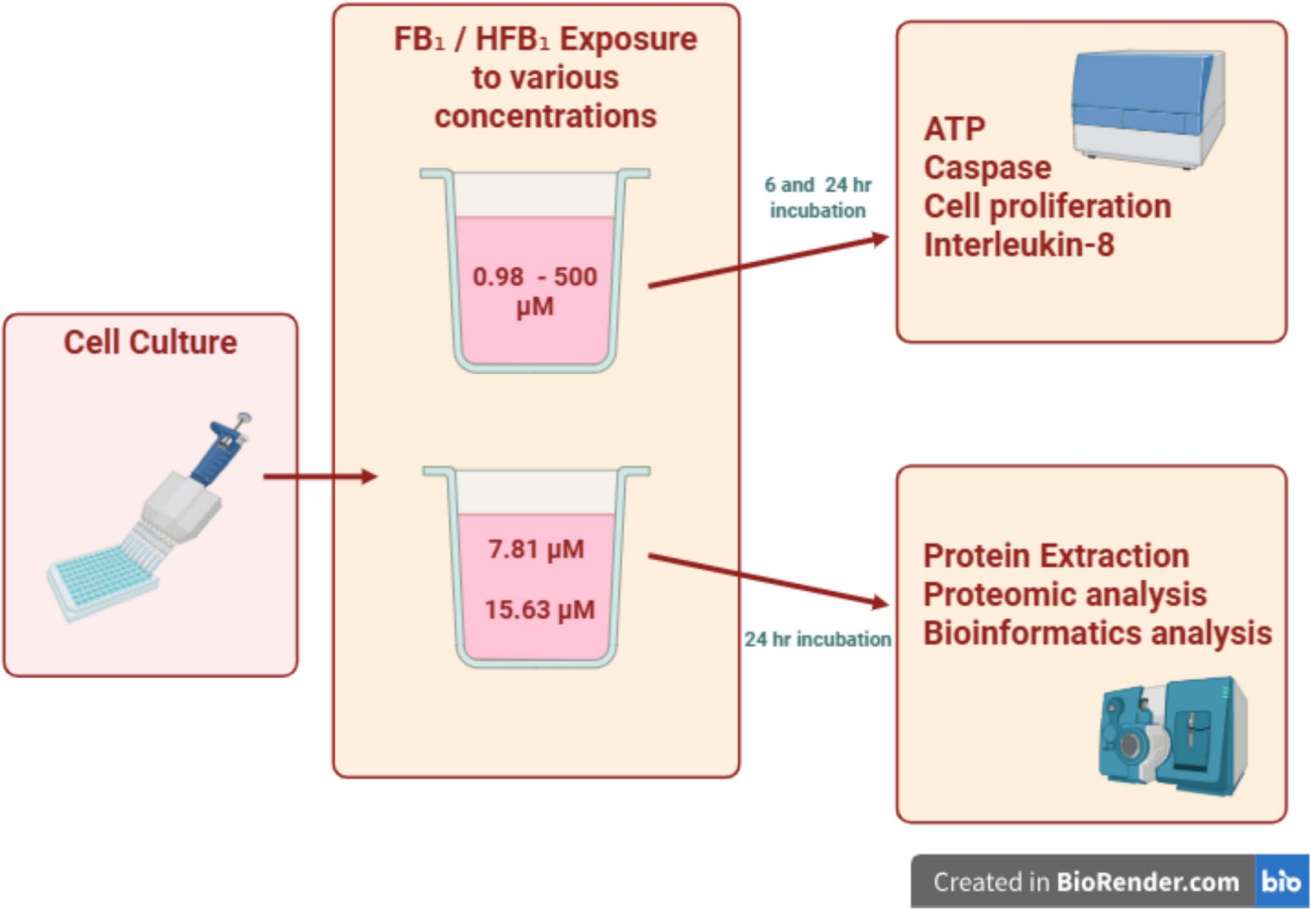
Experimental layout for FB_1_ and HFB_1_ exposure and downstream analysis. The schematic illustrates the experimental design used to assess the effects of FB_1_ and HFB_1_ exposure in IPEC-J2 cell line. Cells were treated with defined concentrations of FB_1_ and HFB_1_, followed by a series of downstream analyses

Non-transformed, secondary intestinal porcine enterocytes (IPEC-J2) were obtained from dsm-firmenich, Tulln, Austria. Cells were inoculated and maintained in DMEM/Ham’s F-12 medium supplemented with heat-inactivated foetal bovine serum (FBS), 1% insulin-transferrin-selenite (ITS), epidermal growth factor (EGF) (Gibco-Life Technologies, Paisley, UK), L-glutamine, and 2-[4-(2-hydroxyethyl)piperazin-1-yl] ethane sulfonic acid (HEPES) buffer (Lonza, Basel, Switzerland). Cells were passaged twice a week at a split ratio of 1:3 and incubated at 37 ℃ in humidified air containing 5% carbon dioxide (CO_2_)/95% air. For the cell optimisation model, upon reaching 70–80% confluency, cells were washed with Hank’s balanced salt solution (HBSS), trypsinated (Lonza, Basel, Switzerland), and seeded in supplemented maintenance medium at the density of 3 × 10^4^ cells per well in 96-well microtitre plates. Cultured cells were incubated for a minimum of 24 h at 37 ℃ in 5% CO_2_/95% air before medium was discarded. Thereafter, cells were exposed to FB_1_ and HFB_1_ concentrations (0.98 µM, 1.95 µM, 3.91 µM, 7.81 µM, 15.63 µM, 31.25 µM, 62.5 µM, 125 µM, 250 µM, and 500 µM) for 6 and 24 h (made up in supplemented DMEM/Ham’s F-12 medium with 0.5% FBS and 1% dimethyl sulfoxide (DMSO)) (Sigma-Aldrich, MO, USA). The control well contained only supplemented DMEM/Ham’s F-12 medium with 0.5% FBS and 1% DMSO (Fig. 1). Each experiment was conducted in five independent experiments and repeated twice.

For the proteomics analysis, cells were seeded in supplemented DMEM/Ham’s F-12 with 10% FBS at the density of 1.5 × 10^6^ cells per dish in 60-mm clear Petri dishes. Thereafter, cells were exposed to FB_1_ and HFB_1_ concentrations (7.81 µM and 15.63 µM) for a period of 24 h under standard incubation conditions (Fig. 1), as described above. Eight replicates were prepared per treatment, resulting in a total of 40 samples. Protein extraction and LC–MS/MS analysis were performed as described below, at the Centre for Proteomic and Genomic Research (CPGR, Cape Town, South Africa).

### FB_1_ and HFB_1_ stock solution preparations

Purified and extracted FB_1_ (> 95%) and HFB_1_ (> 98%) (AMHBI, CPUT, SA) were dissolved in DMSO forming a stock solution of 1 mg/mL, respectively. The optimised model development of FB_1_ and HFB_1_ included concentrations from 0.98 to 500 µM, in supplemented DMEM/Ham’s F-12 medium with 0.5% FBS and 1% DMSO.

For proteomics, a 31.25 μM stock concentration of each toxin was made up in supplemented DMEM/Ham’s F-12 medium with 0.5% FBS and 1% DMSO. The respective stock solutions were further diluted into various concentrations, selected from the optimised model development (7.81 μM and 15.63 μM FB1; 7.81 μM and 15.63 μM HFB_1_). The cells were treated with these sub-cytotoxic levels, insignificantly impairing the overall cell viability (maximum decrease was 20%).

### Cell viability indices and inflammation biomarkers

#### Determination of cell viability (ATP production)

The ATP content of the IPEC-J2 cell line was determined after FB_1_ and HFB_1_ exposure, using the CellTiter-Glo luminescent cell viability kit according to the manufacturer’s instructions (Promega Corporation, Madison, USA). This assay quantifies viable cells based on the amount of intracellular ATP, which is a marker of metabolically active cells. The reagent lyses cells and produces a luminescent signal proportional to ATP levels, indicating cell viability. The signal (expressed as relative light units (RLU)) was recorded using the Veritas microplate luminometer (Turner Biosystems, CA, USA), and data were expressed as a percentage of ATP content of control cells.

#### Determination of apoptosis

After FB_1_ and HFB_1_ exposure, cells were lysed with 100 μL of filtered 0.5% Triton X-100 (Roche, Mannheim, Germany) made up in Dulbecco’s phosphate buffered saline (DPBS) (Lonza, Basel, Switzerland) and stored at − 80 °C until analysis. Caspase-3 and caspase-7 activity was measured using the Caspase-Glo assay kit (Promega Corporation, Madison, USA) according to the manufacturer’s instructions. This luminescent assay detects caspase activity by using a proluminescent substrate that is cleaved by active caspase-3 and caspase-7, releasing a luciferase substrate and generating a luminescent signal proportional to enzyme activity, which indicates apoptosis. The luminescent signal (RLU) was recorded using the Veritas microplate luminometer (Turner Biosystems, CA, USA), and data was expressed as a fold increase relative to the control.

#### Determination of cell proliferation

The cell proliferation ELISA, 5′bromo-2′-deoxyuridine BrdU chemiluminescent assay (Roche, Mannheim, Germany), was used to observe the effect of FB_1_ and HFB_1_ on cell proliferation, according to the assay specifications. This assay measures DNA synthesis by detecting the incorporation of BrdU, a thymidine analogue, into newly synthesised DNA of proliferating cells using an anti-BrdU antibody and a chemiluminescent signal. In a solid black 96-well microtitre plate after FB_1_ and HFB_1_ exposure, chemiluminescence signal (RLU) was detected using the Veritas Microplate Luminometer (Turner Biosystems, CA, USA), and data expressed as a percentage of cell proliferation over controlled cells.

#### Porcine interleukin-8 ELISA

The IL-8 content in the cell supernatant was determined using a porcine IL-8/CXCL8 ELISA kit (R&D Systems, Minneapolis, USA) according to the manufacturer’s instructions. This enzyme-linked immunosorbent assay quantifies IL-8 levels based on the specific binding of antibodies to IL-8, followed by a colorimetric reaction proportional to the cytokine concentration in the sample. After FB_1_ and HFB_1_exposure, cell supernatants (100 μL) were transferred to clear 96-well microtitre plates and stored at − 80 °C until analysis. Absorbance was measured at 450 nm with the Synergy 2 Multimode Microplate Reader (Biotek, Vermont, USA) and data were analysed using the standard curve generated using Gen5 Data Analysis Software (Version 2 for Windows). Extracellular porcine IL-8 was expressed as pg/mL of cell supernatant and as the fold increase relative to the untreated control.

### Statistical analyses

For the statistical analysis of FB_1_ and HFB_1_ concentrations, a one-way analysis of variance (ANOVA) was conducted to determine whether there were statistically significant differences among the experimental groups. This method assessed the overall group effects by comparing the means of each group. The data analysis was performed using GraphPad Prism (version 10.4). In order to further investigate which specific group differences contributed to any significant effects identified by the ANOVA, a post hoc Tukey–Kramer multiple comparison test was applied. This test allowed for pairwise comparisons between group means while controlling for type I error. Statistical significance was considered at a threshold of *p* < 0.05, indicating that differences between groups were deemed significant if the probability of such differences arising by chance was less than 5%.

### Proteomics sample preparation, protein extraction and clean-up

Cells were prepared and treated as described above and harvested after 24-h exposure to FB_1_ and HFB_1_. Medium was discarded and cells were washed with HBSS, trypsinated, and transferred to Eppendorf tubes. The samples were centrifuged for 5 min (500 × g), and the supernatant was discarded. Subsequently, the pellet was washed, centrifuged, the supernatant discarded, and the final pellet placed on ice.

A SIMPLEX protein extraction method by Coman et al. (2016) was modified and used to extract proteins prior to proteomic analysis. Modifications include the addition of acetone (4:1, v/v) overnight instead of methanol (Labchem, Gauteng, SA) for 1 h to the remaining lower phase protein precipitation. Furthermore, protein samples were dissolved in 100 mM tetraethylammonium bromide buffer (TEAB) containing 4 M guanidine hydrochloride (Gu-HCL) and 1% octyl β-D-glucopyranoside (OGP) (Merk/Sigma-Aldrich, Johannesburg, SA). The samples were then placed in a sonicating water bath for 5 min at 4 °C. Cold methanol (800 μL) was added and vortexed for 20 s. This was followed by the addition of chloroform (200 μL) (Merk/Sigma-Aldrich, Johannesburg, SA) and molecular-grade water (600 μL). The samples were vortexed for 20 s after each solvent was added. Samples were then centrifuged at 12,000 × g for 10 min at 4 °C and the top aqueous layer was discarded. An additional 800 μL of methanol was added to the protein layer and vortexed. The samples were then centrifuged for 10 min (12,000 × g) at 4 °C, and the supernatant, discarded. The pellet was left to air dry on ice for 30 min, and samples were stored at − 20 °C until analysis.

### Proteomic analysis

#### Protein solubilisation and quantification

Proteomics was conducted at D-CYPHR, an amalgamation of the proteomics facilitates from the CPGR and UCT IDM, supported by DIPLOMICS. The proteins were solubilised by adding 4% sodium dodecyl sulphate (SDS), 100 mM TEAB and heated to 95 °C for 10 min followed by centrifugation at 10,000 × g for 10 min and the supernatant transferred to a new tube. A sub-volume was removed for protein quantification using the bicinchoninic acid assay (BCA assay) (Merck/Sigma-Aldrich, Johannesburg, SA) according to the manufacturer’s instructions.

#### On-bead HILIC digest

The HILIC magnetic bead workflow was prepared by aliquoting HILIC beads (ReSyn Biosciences, Gauteng, SA) into a new tube and removing the shipping solution. The beads were then washed with 250 μL wash buffer containing 15% acetonitrile (ACN) (Burdick & Jackson, MI, USA) and 100 mM ammonium acetate (pH 4.5) (Merck/Sigma-Aldrich, Johannesburg, SA) for 1 min. The beads were washed again for a total of two washes and resuspended in a loading buffer consisting of 30% ACN and 200 mM ammonium acetate (pH 4.5), to a concentration of 2.5 mg/mL. A total of 20 μg from each sample was transferred to a protein LoBind plate. The protein was then reduced and alkylated by adding 20 mM dithiothreitol (DTT) and 30 mM iodoacetamide (IAA) (Merck/Sigma-Aldrich, Johannesburg, SA), which was followed by a 10-min incubation at 95 °C. Subsequently, HILIC magnetic beads were added at an equal volume to that of the sample at a ratio of 5:1 total protein. The plate was then incubated on a plate shaker (900 × g) for 30 min at room temperature to bind the proteins to the beads. Thereafter, the beads were washed with 500 μL 95% ACN for 1 min, four times. The protein was digested via the addition of trypsin made up in 50 mM TEAB at a ratio of 1:20 total protein and LysC protease (Pierce, MA, USA), added at a ratio of 1:250 total protein. The plate was then incubated on a plate shaker at 45 °C for 2 h. Subsequently, the supernatant containing peptides was removed and dried, and samples were then resuspended in liquid chromatography loading buffer containing 0.1% formic acid (FA) (Merck/Sigma-Aldrich, Johannesburg, SA) and 2.5% ACN.

#### Liquid chromatography-mass spectrometry

Liquid chromatography-mass spectrometry (LC–MS) analysis was conducted with a Q-Exacive quadrupole-Orbitrap mass spectrometer (Thermo Fisher Scientific, MA, USA) coupled with a Dionex Ultimate 3000 nano-ultra-high performance liquid chromatography (UPLC) system. Mass spectrometry was performed using a data-dependent acquisition (DDA) method, specifically a Top10 approach (as indicated by the loop count of 10). The total cycle time was approximately 0.6 s, comprising a 100-ms injection time for the MS1 scan and ten MS2 scans at 50 ms each. Fragmentation was carried out using higher-energy collisional dissociation (HCD), with an AGC target value of 1e5. Additional MS/MS parameters included a resolution of 17,500 (at m/z 200), an isolation window width of 3 m/z, and a normalised collision energy (NCE) of 27%. Data-dependent settings included an underfill ratio of 1%, exclusion of unassigned charges and those with charge states 1, 7, 8, and > 8, peptide match set to ‘preferred’, isotope exclusion enabled, and a dynamic exclusion window of 60 s. The data was acquired using Xcalibur (version 4.1.31.9), Chromeleon (version 6.8- SR13), Orbitrap MS (version 2.9 and build 2926), and Thermo Foundations (version 3.1- SP4). The peptides were dissolved in 0.1% FA and 2% ACN. Thereafter, approximately 400 ng of peptide (injected per sample) was loaded on a C18 trap column (PepMap 100, 300 μM × 5 mm × 5 μM). The samples were then trapped into the column and washed for 3 min with 0.1% formic acid (FA) in LC–MS grade water before the valve was switched and peptides eluted into the analytical column. Chromatographic separation was performed with a Waters nanoEase (Zenfit) m/z peptide charged surface hybrid (CSH) C18 column (75 μM × 25 cm × 1.7 μM). The solvent system used was solvent A consisting of liquid chromatography water and 0.1% FA, and solvent B consisting of ACN and 0.1% FA. The multi-step gradient for peptide separation was generated at 300 nL/min as follows: time change 5 min, gradient change 2–5% solvent B, time change 40 min, gradient change 5–18% solvent B, time change 10 min, gradient change 18–30% solvent B, time change 2 min, gradient change 30–80% solvent B. The gradient was then held at 80% solvent B for 10 min before returning it to 2% solvent B for 15 min. All data acquisition was obtained using Proxeon (Theromo Fisher Scientific, MA, USA) stainless steel emitters. The mass spectrometer was operated in positive ion mode with a capillary temperature of 320 °C and the applied electrospray voltage was 1.95 kV.

### Data analysis for protein identification

The raw data generated was searched against a porcine reference proteome (Pig_Refprot_49792_UP000008227_061221.fasta), downloaded from UniprotKB on 06/12/2021. The raw files were processed using Progenesis QI for Proteomics (Non-linear Dynamics, Newcastle upon Tyne, UK) software and the valid proteins (filtered results to remove reverse hits, common contaminants, and singly charged ions) containing at least two unique peptides were reported. A list of regulated proteins was generated using the same criteria as for valid proteins, with the additional requirement that their *q*-value be less than 0.05. Relative quantification was conducted using Progenesis QI for Proteomics (version 2.0.5556.29015) (Nonlinear Dynamics, Newcastle upon Tyne, UK). The data processing included peak picking, run alignment, and normalisation (singly charged spectra were removed from the processing pipeline). Label-free quantification (LFQ) was performed using Progenesis QI for Proteomics software (Version 2.0, Nonlinear Dynamics, UK) based on data-dependent acquisition (DDA) LC–MS/MS data. In DDA mode, the instrument acquires a full MS1 scan followed by MS/MS (MS2) scans of the most intense precursor ions selected in real time, enabling the identification of a broad range of peptides within a sample. To correct for run-to-run variability, normalisation was applied across samples, and only peptides with high-confidence identifications were included in the quantification process (Li et al. 2009). The data processing steps included peak picking, run alignment, and normalisation. Singly charged ions were excluded to minimise background noise and improve quantification accuracy. Protein quantification was performed using the ‘relative quantitation using non-conflicting peptides’ method, ensuring that only uniquely assigned peptides contributed to the protein-level measurements. The resulting normalised LFQ intensity values were subsequently used for downstream bioinformatics analyses (Cox et al. 2014). Database interrogation was performed with Byonic Software (version 3.8.13) (Protein Metrics, Cupertino, USA) using the *Sus scrofa* (pig) reference proteome mentioned above.

### Bioinformatics analysis

The proteomic meta data obtained were further analysed by bioinformatics analysis to obtain valid proteins and pathways affected by FB1 and HFB_1_ exposure. The statistical analyses were performed in R (version 4.2.2) to assess the significance of the observed fold changes between FB1 and HFB_1_ exposures, and the control groups. Proteins were ranked by raw *p*-value, which was then adjusted for multiple comparisons using the Benjamini–Hochberg (BH) method. The BH procedure was applied to control the false discovery rate (FDR) across multiple hypothesis tests (Benjamini and Hochberg 1995). Two-sample *t*-tests were performed to compare protein abundance between each treatment and control. Significant proteins with adjusted *p*-values < 0.1 and fold-changes ≥ 1.3 or ≤ 0.7 were classified as differentially abundant proteins (DAPs). To visualise the DAPs, volcano plots with log2fold change values on the *x*-axis and − Log10 (BH adjusted *p*-values) on the *y*-axis were plotted using the Enhanced Volcano package in R (version 4.2.2).

To assess relative changes in protein abundance between treatment and control conditions, log₂ fold change (log₂FC) was calculated in R (version 2.2) using the formula: log2FC <—log2(Treatment_Mean/Control_Mean). This log transformation provides a symmetric scale for interpreting both upregulated and downregulated proteins, with positive values indicating upregulation and negative values indicating downregulation. Log₂FC values were then visualised using volcano plots together with BH adjusted *p*-values < 0.1 to identify statistically significant changes. A threshold of ± 0.405 (corresponding to a fold change 1.3) was applied to highlight biologically meaningful differences in either direction. For ease of interpretation, Log₂FC values were also converted back to linear fold change in R, using the inverse function: FoldChange <—2^(log2FC). This transformation allowed DAPs between treatment and control conditions to be expressed in intuitive fold-change terms, complementing the symmetric representation of the log₂ scale. The corresponding log₂FC values are presented in the supplementary Tables S1–S3, and the actual fold changes are discussed in the main text. These steps supported data visualisation and downstream filtering, while preserving the biological interpretability of the observed changes.

### Functional annotation and pathway analyses

Functional annotations for DAPs were carried out by Gene Ontology (GO) to assess the biological processes in R, while using the following packages: DESeq2 and AnnotationDbi packages which ran in the background, clusterProfiler tool package that performed the GO analysis, the org.Ss.eg.db package was used as the porcine database, specific to a certain type of genomic annotation or identifier mapping for *Sus scrofa*, the scientific name for the domestic pig.

### GO and KEGG pathway analysis

Gene Ontology (GO) analysis was performed on the Cytoscape software for further evaluation of the functional enrichment attributes, to annotate the identified proteins in terms of biological process (BP), molecular function (MF), and cellular component (CP) of the identified DAPs. Similarly, Kyoto Encyclopedia of Genes and Genomes (KEGG) enrichment analysis was performed to predict pathways based on the KEGG database. The GO and KEGG pathways were considered significantly enriched if BH adjusted *p*-values were < 0.05.

### Analysis of the protein–protein interaction networks

The STRING (Search Tool for the Retrieval of Interacting Genes/Proteins) database (https://string-db.org) was utilised to retrieve the functional protein–protein interaction (PPI) networks of the identified significant DAPs from each data set separated into specific exposure concentrations (Szklarczyk et al., 2019). All significant DAPs were tested for functional enrichment and network analysis using STRINGApp (via Cytoscape) (Doncheva et al., 2018). Critically, the background set for enrichment was defined as all proteins quantified in the IPEC-J2 proteomic analysis (i.e. the complete list of detected proteins). STRING enrichment uses Fisher’s-exact against the custom input-derived background, which calculates the *p*-value and adjusted *p*-value using the BH method for correction of multiple hypotheses testing. The function categories and pathways with *p*-value < 0.05 were considered significant. Furthermore, additional customisation of the PPI network was developed in the Cytoscape software (version 3.10, http://www.cytoscape.org/) to recognise the target proteins by fold change and clustering methods. The top 50 DAPs with significant PPI were selected for further assessment.

Protein–protein interaction (PPI) networks were constructed using the STRINGApp (STRING database) within Cytoscape. Protein symbols corresponding to differentially abundant proteins (DAPs), meeting criteria of BH adjusted *p*-value < 0.1 and fold-change ≥ 1.3 or ≤ 0.7, were uploaded directly as input (Supplementary Tables S1–S3). A STRING: protein query was used to request the networks, with a confidence (interaction score) cutoff ≥ 0.7. Resulting networks include both experimentally validated and predicted interactions, which were visualised and annotated in Cytoscape with interaction scores and fold-change mapping. Visualisation was customised based on fold-change values (node colour intensity) and connectivity (degree centrality), allowing identification of key hubs or clusters of proteins potentially responsible for cellular responses to FB_1_ and HFB_1_ exposure.

## Results and discussion

### The effect of FB_1_ and HFB_1_ exposure on cell viability

**Fig. 2:**
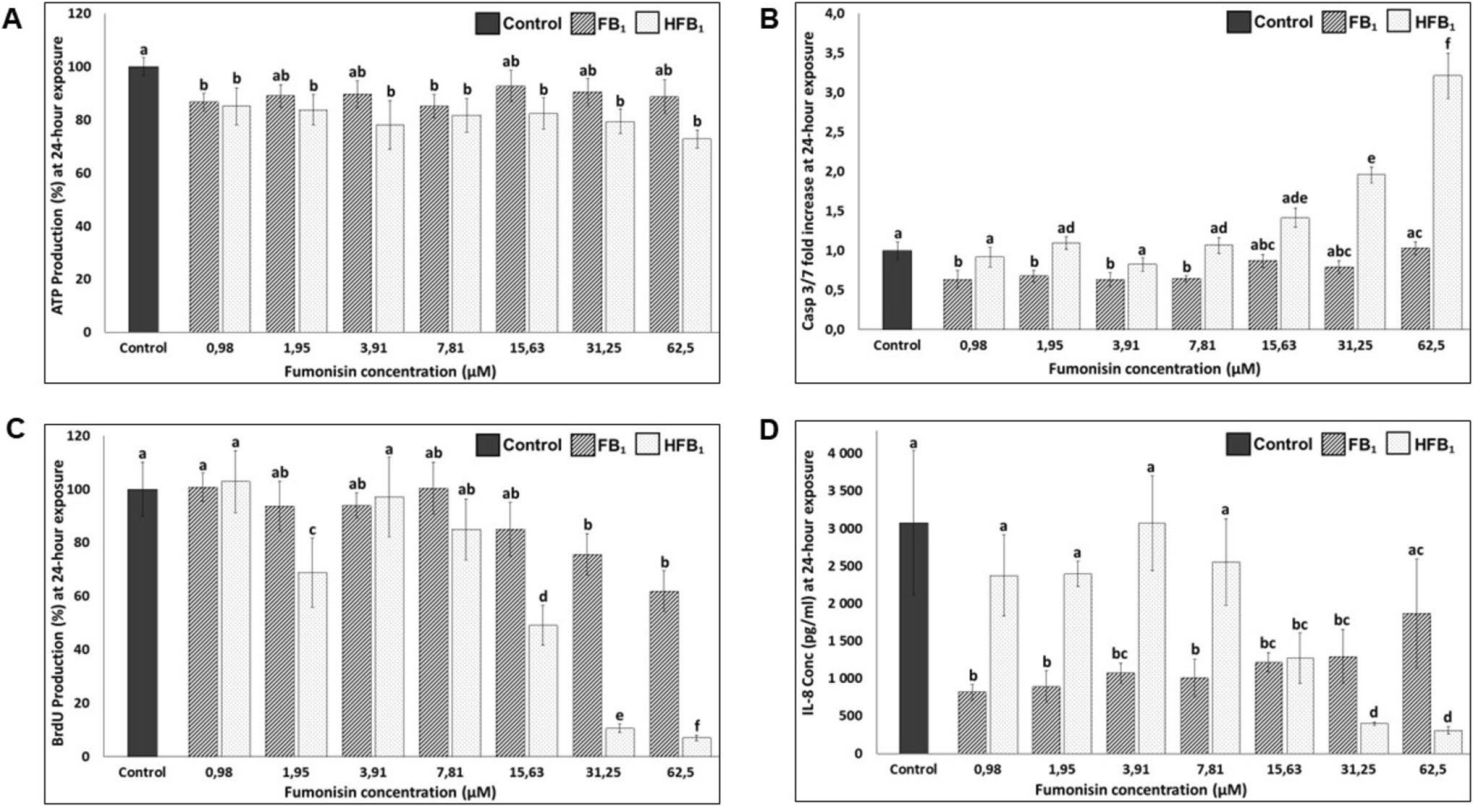
The effect of fumonisin B_1_ (FB_1_) and hydrolysed fumonisin B_1_ (HFB_1_) on (**A**) cell viability (ATP production); (**B**) apoptosis (caspase 3/7-fold increase); (**C**) cell proliferation (BrdU incorporation); and (**D**) an inflammatory biomarker (IL-8: interleukin 8 Conc: concentration) at 24-h incubation periods in IPEC-J2 porcine intestinal cells. ATP-adenosine triphosphate and BrdU production was calculated as a percentage and at a statistical significance of *p* < 0.05. Concentrations include a control and various concentrations of FB_1_ and HFB_1_ (ranging from 0.98 to 500 µM). Statistically significant differences (*p* < 0.05) are shown as lowercase letters above each of the graph bars where each concentration of FB_1_ and HFB_1_ are compared to the control

To establish a concentration-dependent response and select a sub-toxic dose for downstream proteomic analysis, this study first evaluated the effects of FB_1_ and HFB_1_ on IPEC-J2 cell viability across a range of concentrations and time points. This study demonstrated that exposure to FB_1_ at concentrations up to 500 µM did not result in a significant decrease in cell viability in IPEC-J2 cells after 6 (Fig. S1A and S1C) or 24 h (Figs. S1B and 2 A). Notably, FB_1_ at 1.95 µM, 15.63 µM, and 31.25 µM induced a slight but statistically significant increase in cell viability at 6 h (Fig. S1C). After 24 h, exposure to 0.98 µM and 7.81 µM FB_1_ caused a significant decrease in cell viability (Fig. 2A); however, viability remained above 80%. Studies performed by Chen et al. (2019) and Wan et al. (2013) showed that IPEC-J2 cells exposed to FB_1_ for 48 h experienced a significant decline in cell viability at concentrations above 25 µM and 40 µM. Other intestinal cell lines such as the porcine iliac artery endothelial cells were also sensitive to FB_1_ exposure and resulted in a significant decrease in cell viability after 48 h at 50 µg/mL (69 µM) FB_1_ exposure (Yuan et al. 2019). However, 24 h of exposure to various concentrations (1–70 µM) of FB_1_ did not affect cell viability in HT-29 cells and only exhibited a slight decline after 72 h at 70 µM FB_1_ exposure (Minervini et al. 2014). Similarly, many of the mentioned above studies also showed no effect on cell viability after 24 h of FB_1_ exposure. This suggests that the effect of FB_1_ on intestinal cells becomes more cytotoxic dose dependent as time progresses, consistent with findings by Wang et al. (2022), who reported no significant changes in cell viability after 24 h of exposure to 10, 20, and 40 µg/mL FB_1_, but observed a reduction in viability to 95.14% and 83.66% at 20 and 40 µg/mL, respectively, after 48 h.

After 6 h of HFB_1_ exposure, cell viability was significantly increased at concentrations of 0.98 µM, 7.81 µM, 15.63 µM, 31.25 µM, and 250 µM (up to 140%), with a significant decrease observed only at the highest concentration of 500 µM; however, viability remained above 80% (Fig. S1A and S1C). After 24 h of HFB_1_ exposure, a significant decrease in cell viability was observed across concentrations from 0.98 to 62.5 µM, with no significant effect at 125 µM, a significant increase at 250 µM, and a pronounced decrease at 500 µM, where viability was reduced to approximately 40% (Figs. S1B and 2 A). The increase in cell viability at certain concentrations relates to a phenomenon whereby stressed or dying cells release or expose molecules called damage-associated molecular patterns (DAMPs), such as ATP and amphotericin on their surface. In return, it signals the innate immune system and increases inflammation. Extracellular ATP specifically is often released from apoptotic cells where the secretion is associated with the apoptotic stage and type of stress or cell death stimulus (Krysko et al. 2012).

### The effect of FB_1_ and HFB_1_ exposure on apoptosis

Apoptosis was evaluated in response to FB_1_ and HFB_1_ exposure to investigate its potential role in mediating cytotoxic effects and cellular damage, as programmed cell death is a key mechanism underlying toxin-induced tissue injury. An increase in apoptosis was observed in IPEC-J2 cells exposed to 125 µM, 250 µM, and 500 µM FB_1_ after both 6 and 24 h (Fig. S2A and S2B). At lower concentrations, no significant effect on apoptosis was detected after 6 h; however, after 24 h, a decrease in apoptosis was noted at 0.98 µM, 1.95 µM, 3.91 µM, and 7.81 µM compared to control cells (Figs. S2C and 2B). This suggests that cells were undergoing apoptosis from 125 µM exposure independent of the exposure period. Wang et al. (2022) observed a similar finding in IPEC-J2 cells that displayed caspase-3 and caspase-9 cleavage and expression after 48 h from 10 µM up to 40 µg/mL (55 µM) exposure. After 6 h of HFB_1_ exposure, a reduction in apoptosis was observed at 0.98 µM and 3.91 µM compared to control cells, while significant induction of apoptosis occurred at higher concentrations ranging from 62.5 to 500 µM. (Fig. S2A and S2C). The apoptotic response after 24 h of 31.25, 62.51, 125, and 250 µM HFB_1_ exposure exceeded that of 6 h (Figs. S2B and 2B). The rapid decrease in apoptosis at concentrations following 250 µM HFB_1_ after 24 h (Fig. S2B) could be attributed to depleted cell populations in advanced stages of apoptosis. Additionally, cells may also be undergoing a rapid onset of cellular necrosis after a high concentration of HFB_1_ exposure and may not have enough time or energy to initiate apoptotic mechanisms which will therefore not express apoptotic indicators, resulting in lowered apoptosis (Istifli et al. 2019).

### The effect of FB_1_ and HFB_1_ exposure on cell proliferation

To further assess the impact of FB_1_ and HFB_1_ on cellular growth, the effect of exposure to these compounds on cell proliferation was investigated in IPEC-J2 cells. Cell proliferation was not significantly affected after 6 h of exposure to most FB_1_ concentrations, except for a slight inhibitory effect observed at 0.98, 15.36, and 62.5 µM (Fig. S3A and S3C). After 24 h of FB_1_ exposure, concentrations above 31.25 µM resulted in a dose-dependent inhibition of cell proliferation, with greater than 50% inhibition observed at 500 µM (Figs. 2C and S3B). Cell proliferation significantly declined at HFB_1_ concentrations above 62.5 µM following 6 h of exposure (Fig. S3A and S3C), and approximately 90% inhibition at 250 µM and 500 µM. After 24 h of HFB_1_ exposure, cell proliferation significantly declined with an initial decrease observed at 1.98 µM, further inhibition from 15.63 µM, and approximately 90% or greater inhibition at concentrations of 31.25 µM and above (Figs. 2C and S3B). This suggests that, like FB_1_, prolonged exposure of HFB_1_ promotes dose- and time-dependent cytotoxicity. It was observed that after 48-h exposure to FB_1_ concentrations of 25 µg/mL (35 µM) and above, elicits injury to porcine epithelial cells by impeding cell proliferation (Chen et al. 2019). HT-29 cells exposed to FB_1_ concentrations of 8.6 µM and higher also induced a dose-dependent inhibition of cell proliferation after 72 h of exposure (Minervini et al. 2014).

### The effect of FB_1_ and HFB_1_ exposure on IL-8 concentration

The effect of FB_1_ and HFB_1_ exposure on IL-8 concentration was investigated to assess the inflammatory response elicited by these mycotoxins in IPEC-J2 cells. No significant effect on IL-8 concentration was observed at any FB_1_ concentration following 6 h of exposure (Fig. S4A and S4C). A significant decrease in IL-8 concentration was observed following 125 µM, 250 µM, and 500 µM HFB_1_ exposure after 6 h (Fig. S4A and S4C). A significant decrease in IL-8 concentration was observed following FB_1_ exposure at concentrations ranging from 0.98 to 31.25 µM after 24-h exposure, whereas no significant difference was detected at 62.5 µM and higher (Figs. 2D and S4B). HFB_1_ exposure, on the other hand, showed no significant effect on IL-8 concentration at lower concentrations (0.98 to 7.81 µM) after 24 h, whereas concentrations of 15.63 µM and above induced a significant decrease (Figs. 2D and S4B). The decrease in IL-8 correlates with a decrease in cell viability and cell proliferation, and an increase in apoptosis. Interleukin-8 (IL-8) is known as a cell proliferation enhancing cytokine, whereby inflammation is promoted and the control of repair processes during intestinal mucosal injury or cytotoxic stress occurs (Wilson et al. 1999). A study performed using IPEC-1 cells observed a dose-dependent decrease in IL-8 concentration at mRNA and protein levels after 4 days with FB_1_ concentrations up to 72.2 µM (Bouhet et al. 2006). Another study observed no IL-8 response in HT-29 cells after 48-h FB_1_ exposure (Minervini et al. 2014). The current study shows a decrease of IL-8 at all concentrations up to 31.25 µM FB_1_ exposure, which was also observed following 62.5 µM FB_1_ exposure after 24 h (Fig. 2D). These observations coupled with cell proliferation depict that IL-8 was secreted upon FB_1_ exposure beyond 31.25 µM (24 h), affecting an inflammatory response. In an in vivo study performed by Grenier et al. (2012), pigs were fed a HFB_1_-containing feed for 14 days and the study reported that the proximal section of the intestine (where IPEC-J2 cells originate from) produced a higher concentration of IL-8 compared to FB_1_. In the current study, a similar trend was observed at concentrations up to 7.81 µM HFB_1_ after 24 h of exposure (Fig. 2D). However, an increase in IL-8 promotes a decrease in the cell survival rate (Qazi et al. 2011). This suggests that HFB_1_ may be metabolised differently in the gut in vivo compared to in vitro, where systemic factors such as host enzyme activity or gut microbiota could modify HFB_1_’s biological activity, resulting in immune responses that are not replicated in isolated epithelial cell models.

### Identification of differentially abundant proteins by liquid chromatography-mass spectrometry

To investigate the molecular mechanisms and pathways of proteins influenced by FB_1_ and HFB_1_ on the IPEC-J2 cell line, a comparative proteomic analysis was performed. Proteins are the foundation of cellular functions, whereby alterations of key proteins would directly affect cellular outputs (McArdle and Menikou 2020). Cells were exposed to 7.81 µM and 15.63 µM FB_1_ and HFB_1_, respectively, for proteomic analysis. These concentrations were selected as they are sub-cytotoxic, as demonstrated by prior assessments of cell viability (Fig. 2A) and caspase-3/7 activity (Fig. 2B), which showed no significant loss in viability or induction of apoptosis at these levels. Specifically, these concentrations represent the highest doses at which no statistically significant cytotoxic or apoptotic effects were observed, thereby allowing for the detection of early molecular responses without confounding cell death-related effects. This approach ensures that the observed proteomic alterations are reflective of subtle cellular stress or early-stage responses to FB_1_ and HFB_1_ exposure, rather than downstream effects of overt toxicity. The intracellular proteins of cells exposed to FB_1_ and HFB_1_ as well as the untreated control group were quantified by LC–MS/MS methods. The proteins with a fold change ≥ 1.3 and ≤ 0.7 (mean value of all compared groups) and *p*-value (*t*-test of all comparison groups) < 0.1 by comparing each treated group to the control group were defined as the significantly differentially abundant proteins (DAPs) (Table 1). Differential expression analysis of the protein datasets generated the lists of significant DAPs, fulfilling the selection criteria of fold change ≥ 1.3 and ≤ 0.7, and BH adjusted *p*-value < 0.1. Fold change thresholds ≥ 1.3 and ≤ 0.7 for the selected upregulated and downregulated proteins, respectively, were selected in this study because the more commonly applied FC threshold of 1.5-fold was not sensitive enough. The application of FC threshold for this study permitted identification of a larger number of proteins whose differential expression from controls was statistically significant, allowing a better opportunity to identify enriched pathways. The selected threshold is less stringent and may include moderate changes; however, this cut-off was chosen based on precedent in similar proteomic analyses where moderate changes were also of interest, to balance sensitivity and specificity in detecting biologically relevant protein alterations (Black et al. 2021; Li et al. 2024).

**Table 1:** The top 52 DAPs between FB_1_ and HFB_1_ compared to the control. Fold change (FC) values in bold represent upregulated proteins > 1.3; values ≥ 1.3 indicate upregulation; and values ≤ 0.7 indicate downregulation

	**Protein symbol**	**Protein name**	**HFB**_**1**_** (7.81 µM)**		**HFB**_**1**_** (15.63 µM)**		**FB**_**1**_** (15.63 µM)**	
			**Adjusted *p*-value**	**FC**	**Adjusted *p*-value**	**FC**	**Adjusted *p*-value**	**FC**
1	CDK6	Cyclin-dependent kinase 6	0.056	**1.637**				
2	ARFGEF1	ADP ribosylation factor guanine nucleotide	0.047	**1.581**				
3	ACTN2	Actinin alpha 2	0.081	**1.531**				
4	FTL	Ferritin light chain	0.015	**1.500**	0.003	**1.496**		
5	CEP192	Centrosomal protein	0.057	**1.458**				
6	VEZT	Vezatin, adherens junctions transmembrane protein	0.075	**1.424**				
7	MRPL18	Mitochondrial ribosomal protein	0.066	**1.416**				
8	TBC1D23	TBC1 domain	0.077	**1.377**				
9	GCA	Grancalcin	0.059	**1.370**				
10	PGPEP1	Pyroglutamyl-peptidase I	0.046	**1.359**				
11	GOLT1B	Golgi transport 1B	0.083	**1.355**				
12	FTH1	Ferritin heavy chain	0.031	**1.350**	0.004	**1.535**		
13	PDCD10	Programmed cell death protein	0.047	**1.347**				
14	MRPL28	Mitochondrial ribosomal protein	0.049	**1.343**				
15	ABRACL	ABRA C-terminal like	0.072	**1.337**				
16	RPS21	40S ribosomal protein S21	0.057	0.754				
17	CYB5B	Cytochrome b5 heme-binding	0.071	0.752				
18	NFU1	NFU1 iron-sulphur cluster	0.058	0.745				
19	SKP1	S-phase kinase	0.082	0.740				
20	CD44	CD44 molecule	0.058	0.732				
21	WDR48	WD repeat domain	0.045	0.724				
22	TINAGL1	Tubulointerstitial nephritis antigen like 1	0.004	0.716	0.032	0.739		
23	AGRN	Agrin	0.055	0.699				
24	NTN4	Netrin 4	0.048	0.698	0.001	0.549		
25	ATP5F1D	ATP synthase subunit delta	0.045	0.694				
26	RPS28	40S ribosomal protein	0.028	0.690				
27	S100A11	Protein S100-A11	0.050	0.683				
28	SRBD1	S1 RNA binding domain 1	0.032	0.677				
29	SH3BGRL3	SH3 domain-binding glutamic acid	0.048	0.663				
30	FN1	Fibronectin 1	0.003	0.647	0.000	0.585	0.049	**1.371**
31	PPP1R2	Protein phosphatase 1 regulatory inhibitor subunit	0.087	0.642				
32	INTS3	Integrator complex subunit 3	0.094	0.639				
33	SDC4	Syndecan-4	0.005	0.629	0.001	0.623		
34	ST14	Suppressor of tumorigenicity 14	0.006	0.613	0.004	0.586		
35	CLNS1A	Chloride nucleotide-sensitive	0.044	0.607				
36	SPARC	Secreted protein acidic and cysteine rich	0.054	0.605	0.093	0.575		
37	CD276	CD276 molecule	0.073	0.599			0.052	**1.469**
38	FBLN2	Fibulin-2	0.003	0.573	0.000	0.547		
39	CHCHD2	Coiled-coil-helix	0.055	0.469				
40	CCN3	Cellular communication	0.078	0.433				
41	RBP4	Retinol-binding protein					0.056	**1.707**
42	TGFBR2	TGF-beta receptor type-2					0.053	**1.378**
43	EHD2	GTPase superfamily					0.006	**1.335**
44	ATF3	Cyclic AMP-dependent transcription factor ATF-3			0.001	**1.368**		
45	LAMC1	Laminin subunit gamma-1			0.032	0.753		
46	CXADR	CXADR Ig-like cell adhesion molecule			0.013	0.750		
47	QSOX1	Quiescin sulfhydryl oxidase			0.000	0.727		
48	CCN1	Cellular communication			0.006	0.723		
49	MET	Hepatocyte growth factor receptor			0.053	0.672		
50	ITM2B	Integral membrane protein 2B			0.033	0.666		
51	TFRC	Transferrin			0.000	0.628		
52	CDH6	Cadherin 6			0.070	0.447		

**Fig. 3:**
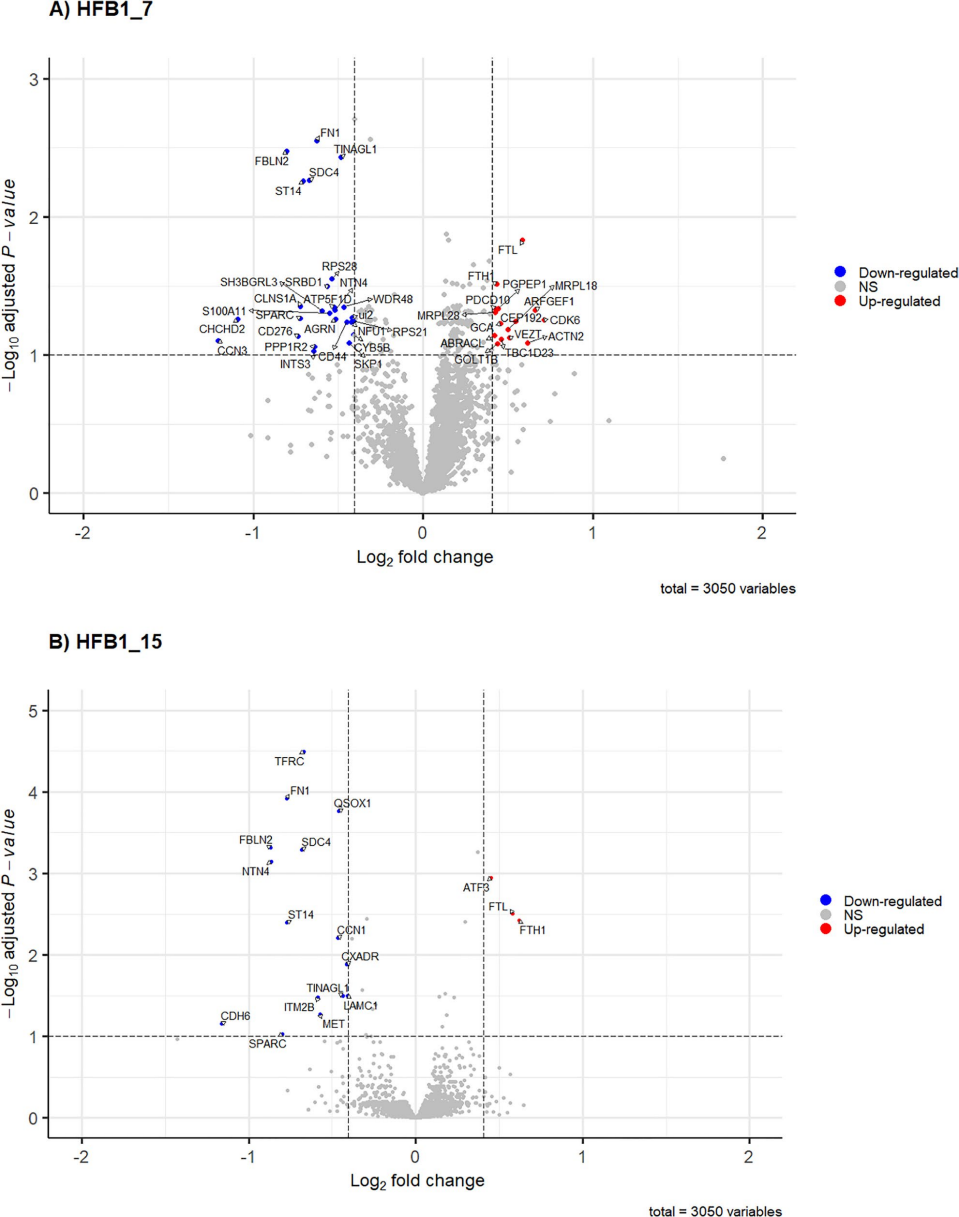

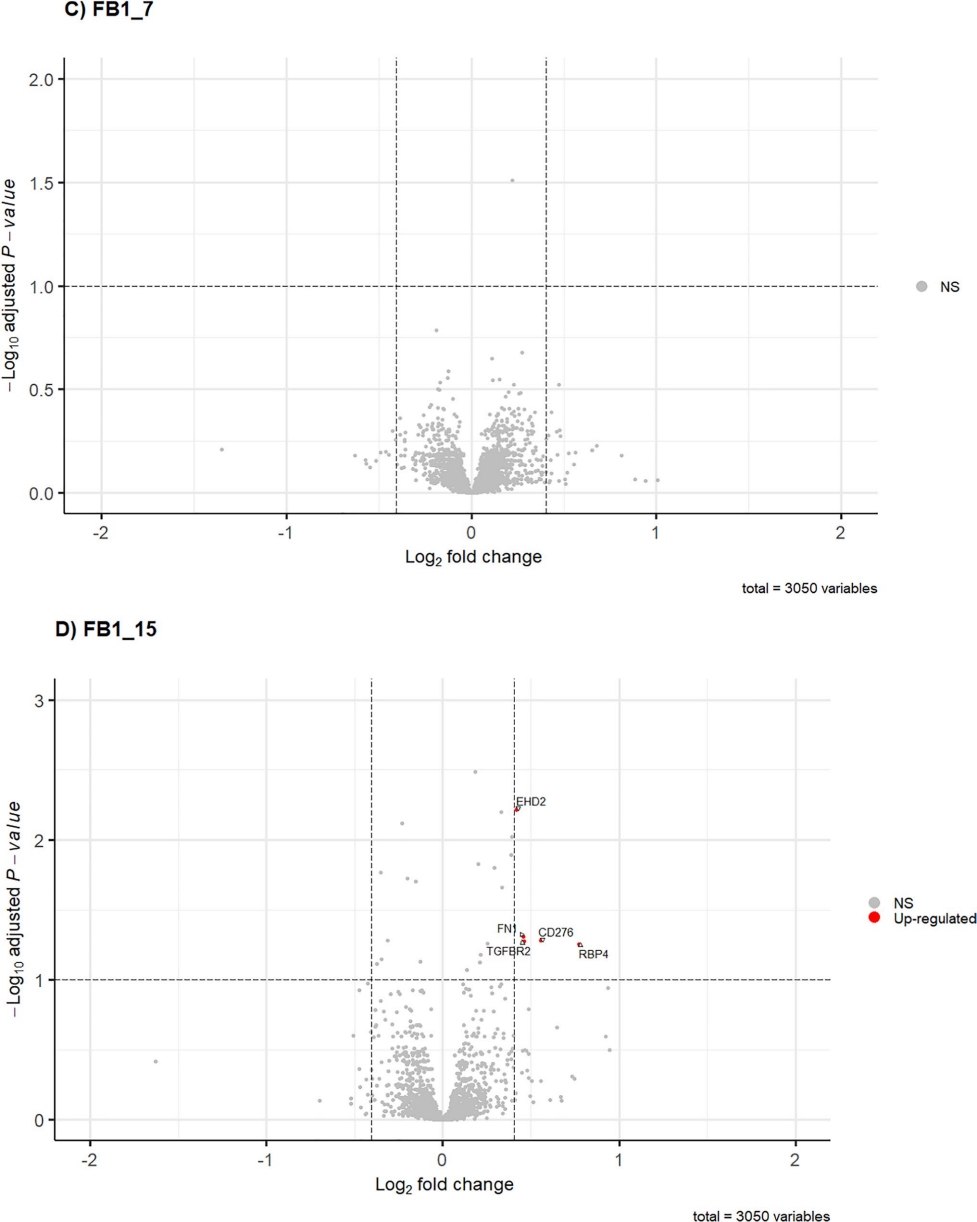
Effect of HFB_1_ (**A**, **B**) and FB_1_ (**C**, **D**) on DAPs, both at the different concentrations 7.81 µM (represented as HFB1_7 and FB1_7) and 15.63 µM (represented as HFB1_15 and FB1_15), in the intestinal porcine cell line IPEC-J2, respectively. The colours indicate threshold of significant proteins which contain BH adjusted *p*-values ≤ 0.1, and fold-change ≥ 1.3 or ≤ 0.7. Significant downregulated proteins are shown in blue, upregulated are shown in red, whereas non-significant proteins are shown in grey

The list of total DAPs along with the FC values and BH-adjusted *p*-values are provided in Table 1. These DAPs for all datasets are graphically represented using the volcano plots in Fig. 3A–D.

A total of 52 DAPs were generated from FB_1_- and HFB_1_-exposed cells compared to the control (unexposed cells) (Table 1). A total of 40 DAPs were identified after 7.81 µM HFB_1_ exposure, comprising 15 upregulated and 25 downregulated proteins. In contrast, exposure to 15.63 µM HFB_1_ resulted in the upregulation of 3 proteins and downregulation of 15 proteins (Table 1; Fig. 3A and 3B). Cells that were exposed to 15.63 µM FB_1_ resulted in five upregulated DAPs and none that were considered significant following 7.81 µM FB_1_ exposure (Table 1; Fig. 3C and 3D).

**Fig. 4:**
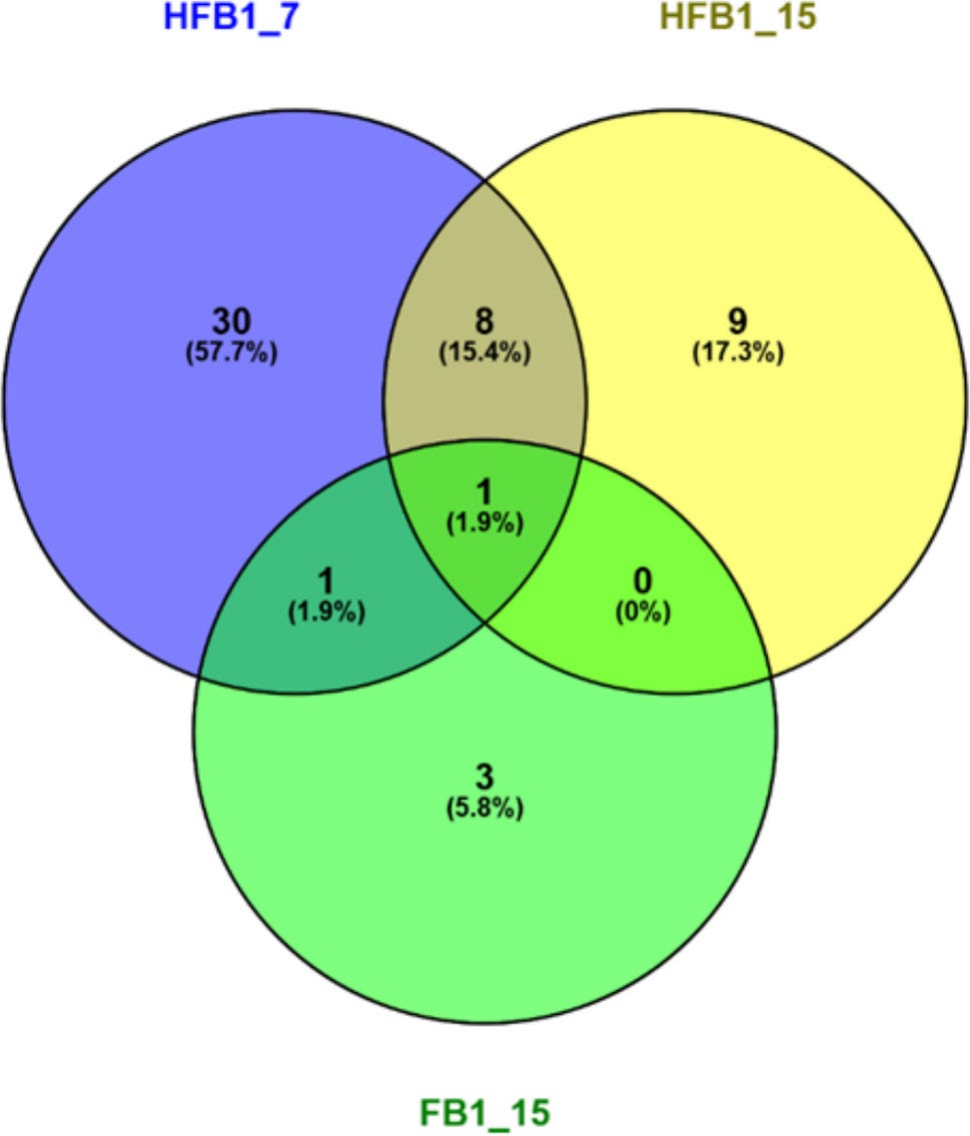
Venn diagram of differentially abundant proteins (DAPs) identified in 7.81 µM HFB_1_ (represented as HFB1_7), 15.63 µM HFB_1_, and 15.63 µM FB_1_ represented as HFB1_15 and FB1_15) exposure groups

The Venn diagram (Fig. 4) illustrates the overlap and uniqueness of DAPs identified under three exposure conditions: 7.81 µM HFB_1_, 15.63 µM HFB_1_, and 15.63 µM FB_1_. A total of 40, 18, and 5 DAPs were identified in the 7.81 µM HFB_1_, 15.63 µM HFB_1_, and 15.63 µM FB_1_ groups, respectively. FN1 was the only DAP common to all three conditions, suggesting its potential role as a universal biomarker or core responder to both compounds. Eight additional proteins (FBLN2, TINAGL1, SDC4, ST14, FTL, FTH1, NTN4, and SPARC) were shared between the two HFB_1_ concentrations, indicating a conserved proteomic response specific to HFB_1_ exposure. In contrast, FB_1_ showed minimal overlap with HFB_1_, sharing only FN1 and CD276, and displayed a largely distinct DAP profile. The highest number of unique proteins was observed in the 7.81 µM HFB_1_ group, suggesting a broader cellular response at this concentration.

Overall, HFB_1_ exposure resulted in a more extensive alteration of the cellular proteome compared to FB_1_ under the tested in vitro conditions. A subset of proteins commonly regulated by both FB_1_ and HFB_1_ will be discussed in detail, while unique proteins associated with specific concentrations will be briefly highlighted.

### Functional enrichment analysis of DAPs in FB_1_ and HFB_1_ related to Gene Ontology

The functional enrichment analysis of differentially abundant proteins (DAPs), performed using the porcine genome annotation database, identified distinct biological processes that appear to be altered in response to HFB_1_ exposure. The STRING enrichment analysis was performed using the entire *Sus scrofa* proteome as the background database. While this is the standard approach in many studies, it may introduce bias due to differences between the specific cell line proteome and the broader species proteome. Ideally, a cell line–specific proteome should be used as a control to better reflect the expressed protein background and improve the accuracy of enrichment results. However, such a database was not available for the cell line used in this study. Therefore, the identified enrichments should be interpreted with caution, as some observed alterations may reflect general differences between the cell line and the species-level proteome rather than specific biological changes. No significantly downregulated DAPs were found following exposure to 15.63 µM FB_1_, and no significant Gene Ontology (GO) terms were enriched for FB_1_-treated cells at either concentration. In contrast, exposure to 7.81 µM HFB_1_ led to the downregulation of proteins associated with Golgi apparatus function and ribosomal processes, while 15.63 µM HFB_1_ downregulated proteins involved in ribonucleoside monophosphate biosynthesis, metabolism, and negative regulation of the ERK1/ERK2 cascade (Supplementary Fig. S5). These processes are fundamental to protein synthesis, intracellular transport, and signalling regulation, suggesting that HFB_1_ impairs essential cellular functions. Furthermore, HFB_1_ exposure at both concentrations upregulated proteins associated with extracellular matrix (ECM) binding, protein complex binding, and morphogenesis of branching epithelium. These GO terms are typically linked to tissue remodelling and structural reorganisation. Based on these associations, the enrichment of such processes points towards indicative of an adaptive cellular response to HFB_1_-induced stress, potentially involving cytoskeletal rearrangement or ECM remodelling. The absence of enriched GO terms in the FB_1_ groups emphasises a comparatively lower degree of pathway-level disruption under the tested conditions.

### Functional enrichment analysis of DAPs in FB_1_ and HFB_1_ related to KEGG pathways

**Fig. 5:**
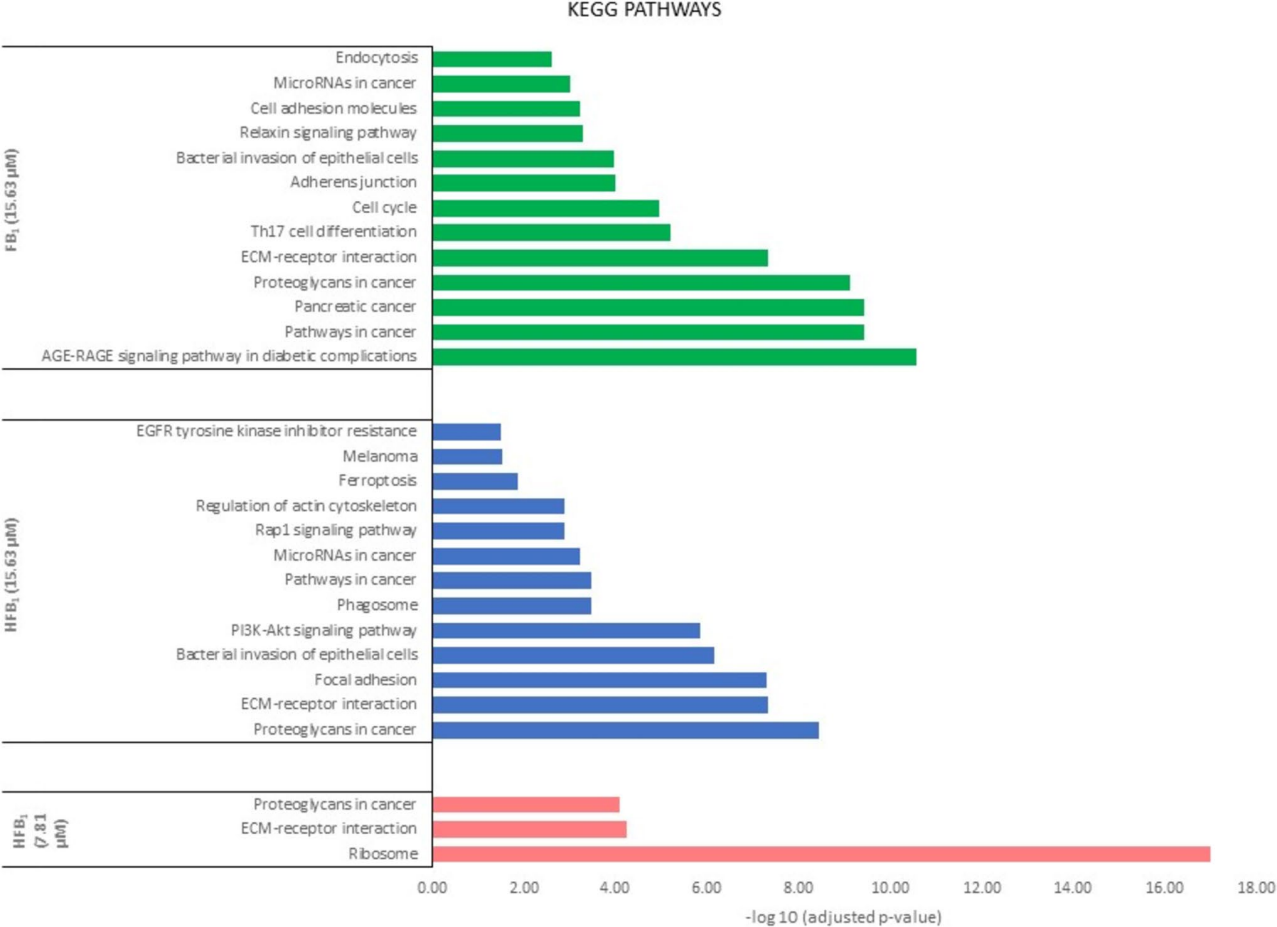
Enrichment analyses of KEGG pathways of the significant enriched proteins associated with 15.63 µM FB_1_, 15.63 µM HFB_1_ (represented as FB1_15 and HFB1_15) and 7.81 µM HFB_1_ (represented as HFB1_7) exposed IPEC-J2 cells. All terms are ranked by adjusted *p*-value

The KEGG pathway enrichment analysis (Fig. 5) illustrates the most significantly affected pathways in response to different treatments based on adjusted *p*-values.

KEGG pathway enrichment analysis revealed differential modulation of several signalling pathways across treatment groups. In the FB_1_ (15.63 μM) exposure group, pathways associated with inflammation, cell adhesion, and cancer progression, such as ECM-receptor interaction, proteoglycans in cancer, and the AGE-RAGE signalling pathway, were significantly enriched (green bars). The HFB_1_ (15.63 μM) exposure also showed enrichment in cancer-related and immune signalling pathways, including the PI3K-Akt signalling pathway, focal adhesion, and phagosome pathways (blue bars). Notably, the HFB_1_ (7.81 μM) exposed group demonstrated a more focused enrichment pattern, with significant involvement of ribosomal activity and cancer-associated ECM-receptor interactions (pink bars). These findings suggest dose- and formulation-dependent modulation of key cellular pathways involved in inflammation, adhesion, and tumorigenesis.

### Shared DAPs in IPEC-J2 cells after FB_1_ and HFB_1_ exposure

Among all concentrations, Fibronectin 1 (FN1) was the only protein consistently affected by exposure to 15.63 µM FB_1_ and both concentrations of HFB_1_(Table 1; Fig. 4). FN1 is crucial for cellular integrity, wound healing, and intestinal epithelial response to injury (Kolachala et al. 2007; Niederlechner et al. 2012; Sun et al. 2020). In previous studies, FN1 levels were reduced during intestinal injury, correlating with increased apoptosis (Niederlechner et al. 2012). Similarly, in our study, FB_1_ upregulated FN1, while HFB_1_ downregulated it, indicating that HFB_1_ exposure may induce more severe intestinal injury. FN1 has also been linked to tumour progression and poor prognosis in various cancers (Cai et al. 2018; Lou et al. 2013; Nakagawa et al. 2014; Sponziello et al. 2016; Sun et al. 2020; Waalkes et al. 2010; Wang et al. 2017; Xiao et al. 2018), and its upregulation in FB_1_-exposed cells may explain the carcinogenic effects observed in animal models (Gelderblom et al. 1993). Our findings align with previous reports demonstrating the oncogenic role of FN1, which was shown to be upregulated in gastric cancer (GC) tissues and cell lines, correlating with increased tumour invasion, advanced TNM stage, lymph node metastasis, and poor patient prognosis (Zhou et al. 2020). In this study, FB_1_ exposure led to an upregulation of FN1, potentially supporting its pro-tumorigenic effect, while HFB_1_ exposure resulted in FN1 downregulation, suggesting a divergent and possibly less oncogenic cellular response. This contrasting regulation highlights a key molecular difference between FB_1_ and HFB_1_ that may underlie their distinct biological effects.

Proteomic analysis additionally revealed one shared DAP following FB_1_ and 7.81 µM HFB_1_ exposure that are known to be involved in cellular processes relevant to cancer biology. CD276 (B7-H3), an immune checkpoint molecule, was upregulated following 15.63 µM FB_1_ exposure (Table 1; Fig. 4). Elevated CD276 expression has been associated with immune evasion and poor cancer prognosis (Liu et al. 2021), suggesting that FB_1_ may influence immune modulation. Conversely, CD276 was downregulated following 7.81 µM HFB_1_ exposure, potentially indicating a different cellular response.

### Distinct upregulated and downregulated DAPs in FB_1_ and HFB_1_ exposure

Despite the two shared DAPs, several distinct DAPs were uniquely affected by either FB_1_ or HFB_1_, indicating compound-specific proteomic responses. The distinct upregulated proteins at 15.63 µM FB_1_ included retinol-binding protein 4 (RBP4), TGF-β receptor type 2 (TGFBR2), and GTPase superfamily (EHD2) (Table 1; Fig. 4). RBP4, a protein involved in vitamin A transport (Perduca et al. 2018), was significantly upregulated following FB_1_ exposure. Dysregulation of RBP4 has been linked to metabolic and inflammatory disturbances in the liver, pancreas, and vascular system, which may relate to the known adverse effects of FB_1_ on these organs. TGFBR2, a key receptor in the TGF-β signalling pathway that regulates cell proliferation and apoptosis (Fynan and Reiss 1993; Kim 2001; Markowitz and Roberts 1996), was upregulated by FB_1_. While normally acting as a tumour suppressor, dysregulated TGFBR2 expression in hepatocellular carcinoma (HCC) has been associated with enhanced tumour progression through mechanisms such as epithelial-to-mesenchymal transition and metastasis (Hao et al. 2019). Similarly, EHD2, involved in membrane remodelling and lipid homeostasis (Shah et al. 2014; Simone et al. 2014; Zhang et al. 2022), was also upregulated by FB_1_, potentially contributing to HCC development by promoting altered lipid metabolism and membrane dynamics that facilitate cancer cell survival, motility, and invasiveness. Together, the increased expression of TGFBR2 and EHD2 may reflect pathways implicated in HCC pathogenesis.

Between 7.81 µM and 15.63 µM HFB_1_, a total of eight DAPs were identified, of which two were upregulated and six were downregulated (Table 1; Fig. 4). The upregulation of ferritin light (FTL) and heavy chains (FTH1) after exposure to both concentrations of HFB_1_ suggests altered iron homeostasis, which has been linked to increased oxidative stress and tumour-promoting environments in some contexts (Grazi et al. 1995). The downregulation of netrin-4 (NTN4), a molecule implicated in cell adhesion and migration (Esseghir et al. 2007; Latil et al. 2003), and ST14, a serine protease with known tumour suppressor functions in the gastrointestinal tract (Kosa et al. 2012; Danielsen et al. 2018), further suggests HFB_1_ may affect pathways involved in epithelial integrity and regulation. The downregulation of SPARC by HFB_1_, a matricellular protein associated with tissue remodelling and tumour progression (Brabender et al. 2003; Framson and Sage 2004; Schiemann et al. 2003; Takemasa et al. 2001; Wang et al. 2004), may reflect disrupted extracellular matrix dynamics. Similarly, decreased expression of TINAGL1, FBLN2, and SDC4 suggests potential perturbations of HFB_1_ in angiogenesis and wound-healing pathways (Bernfield et al. 1992; Pap and Bertrand 2013). These protein changes do not imply direct oncogenic effects of HFB_1_, but may represent early alterations in cell signalling, stress response, or extracellular matrix interaction that warrant further investigation.

Exposure to 73.81 µM HFB_1_ resulted in 30 distinct significantly regulated DAPs, including both upregulated and downregulated proteins (Table 1; Fig. 4). The most upregulated proteins following exposure to 7.81 µM HFB_1_ were CDK6, ARFGEF1, and ACTN2, suggesting an early cellular stress response affecting cell cycle regulation, membrane trafficking, and cytoskeletal dynamics. CDK6, a key regulator of the G1 phase of the cell cycle, has been implicated in DNA damage and apoptotic signalling (Chu et al. 2020; Scheicher et al. 2015). The upregulation of ARFGEF1, a protein involved in maintaining Golgi structure and vesicle trafficking, may reflect disruptions in intracellular transport and neuronal function (Shen et al. 2007; Xu et al. 2022; Zhou et al. 2013). ACTN2, primarily expressed in muscle tissue but also linked to integrin-mediated signalling pathways, is involved in actin filament organisation and cell motility (Beggs et al. 1992; Burridge and McCullough 1980; Lindholm et al. 2021). Its increased expression may indicate cytoskeletal rearrangement and integrin activation. Collectively, the upregulation of these proteins may represent a coordinated cellular response to HFB_1_ exposure, potentially involving alterations in cell cycle control, membrane integrity, and cytoskeletal function. During exposure to 15.63 µM HFB_1_, ATF3 was significantly upregulated, suggesting activation in response to cellular stress signals such as DNA damage and oxidative stress, which may contribute to the observed increase in cell death and inflammation (Hai et al. 1999, 2010; Zhou et al. 2017).

At 7.81 µM HFB_1_, significantly downregulated proteins included CCN3 and CHCHD2 (Table 1; Fig. 4). CCN3 is involved in angiogenesis and inflammation (Jun and Lau 2011), while CHCHD2 regulates mitochondrial function and apoptosis, potentially contributing to the observed increase in apoptotic activity (Akiyama et al. 2017; Liu et al. 2015). At 15.63 µM HFB_1_, CDH6, TFRC, and ITM2B were significantly downregulated. CDH6, a cadherin glycoprotein involved in morphogenesis of the CNS and kidneys (Cho et al. 1998; Zhao et al. 2021), may be linked to tumour progression when dysregulated. ITM2B, known for its role in apoptosis and neurodegenerative disorders, may reflect impaired apoptotic signalling or cancer-related pathways (Baron and Pytel 2017; Xian et al. 2024). Downregulation of TFRC, essential for iron uptake and DNA synthesis, suggests disrupted iron homeostasis, which has been associated with enhanced tumour progression and altered cell metabolism (Gammella et al. 2017; Forciniti et al. 2020; Marchese et al. 2016). Together, these changes in protein expression indicate that HFB_1_ may disrupt key cellular processes, including apoptosis, iron regulation, and developmental signalling, that contribute to cytotoxicity and potentially initiate or promote tumourigenic pathways.

### Protein–protein interaction (PPI) network and pathway analysis of DAPs in FB_1_ and HFB_1_

To identify key proteins involved in the cellular response to FB_1_ and HFB_1_ exposure, protein–protein interaction (PPI) networks were constructed using the STRING database based on the DAPs identified following exposure of the cells. A high-confidence threshold (score ≥ 0.7) was applied to select PPIs, and the data were exported to Cytoscape for further analysis. The selection criteria were based on datasets originating from *Sus scrofa*, the porcine model, which is relevant to the context of the study.

**Fig. 6:**
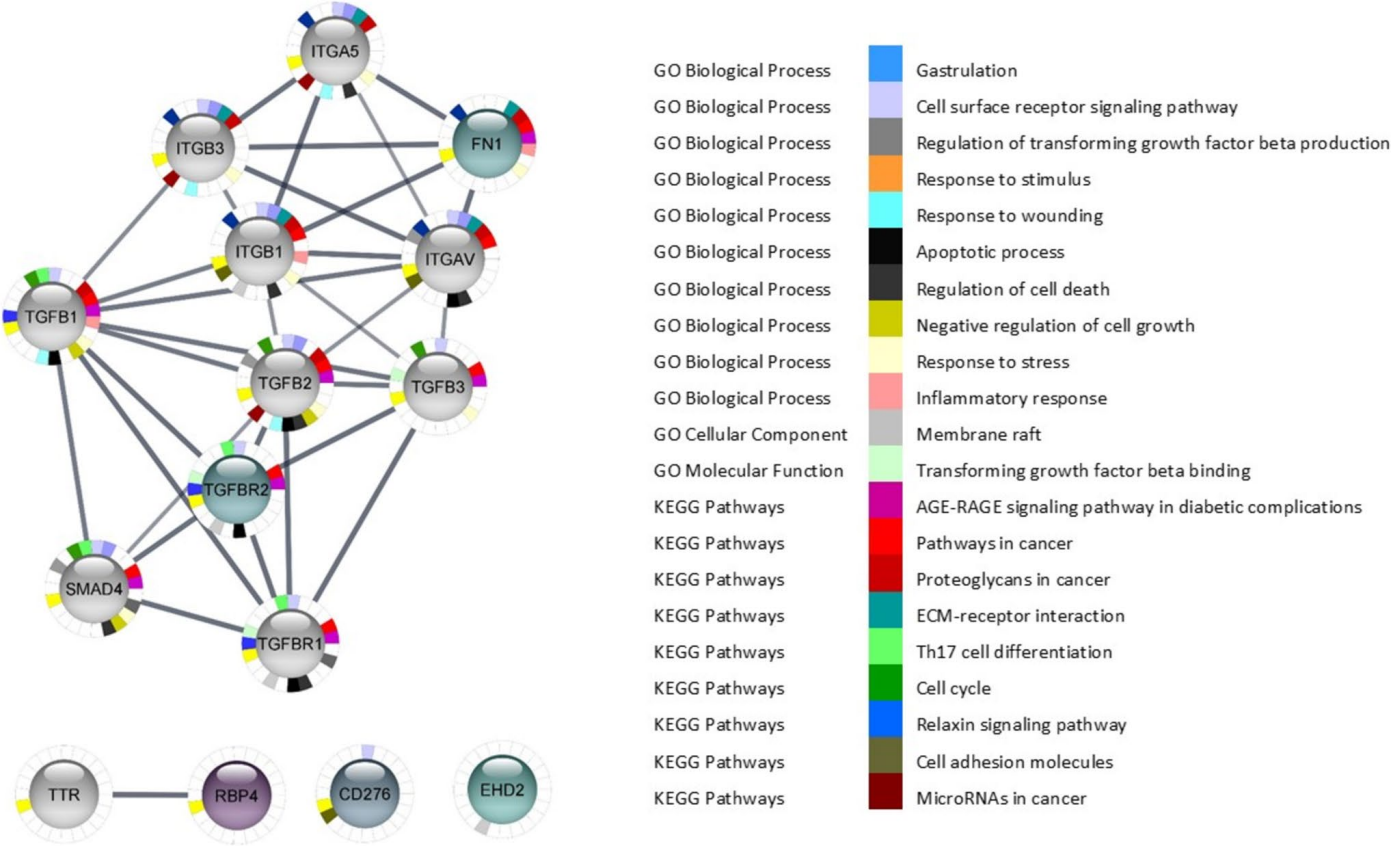
PPI network of 15.63 µM FB_1_ to determine the signalling pathways of the five significant upregulated proteins in darker green to purple. The PPI network consisted of 15 nodes and 32 edges

By identifying common proteins across the different exposure concentrations, we were able to highlight both shared and unique pathways affected by FB_1_ and HFB_1_. In particular, FN1 was upregulated following exposure to 15.63 µM FB_1_. Using the STRING database, it was observed that FN1 has strong predicted interactions with several integrin subunits, including integrin subunit alpha 5 (ITGA5), integrin subunit alpha V (ITGAV), integrin subunit beta 1 (ITGB1), and integrin subunit beta 3 (ITGB3) (Fig. 6). Although these interactions were not directly identified in our experimental dataset, they provide mechanistic context to the observed FN1 upregulation. This supports a possible involvement of integrins in promoting cellular adhesion, migration, and differentiation. Furthermore, this aligns with previous studies reporting that integrin-FN1 signalling plays a pivotal role in tumour progression, particularly in epithelial cancers (Deng et al. 2019; Li et al. 2014; Desgrosellier and Cheresh 2010).

FB_1_ exposure enriched cancer-related pathways, such as proteoglycans in cancer and ECM-receptor interaction, which are implicated in cellular signalling, adhesion, and tissue remodelling (Desgrosellier and Cheresh 2010). The integrin-related pathways induced by FB_1_ exposure suggest its potential role in tumour progression, angiogenesis, and cell migration via transforming growth factor-beta (TGF-β) signalling (Mostafavi-Pour et al. 2018; Ren et al. 2009). Furthermore, our results are consistent with previous studies indicating that FB_1_ exposure affects the matrix cell organisation, as observed in the jejunum through integrin and actin pathways (Dopavogui et al. 2022). The findings suggest that FB_1_ exposure contributes to tumour progression by promoting these key cellular processes, including inflammation and the suppression of cell proliferation.

**Fig. 7:**
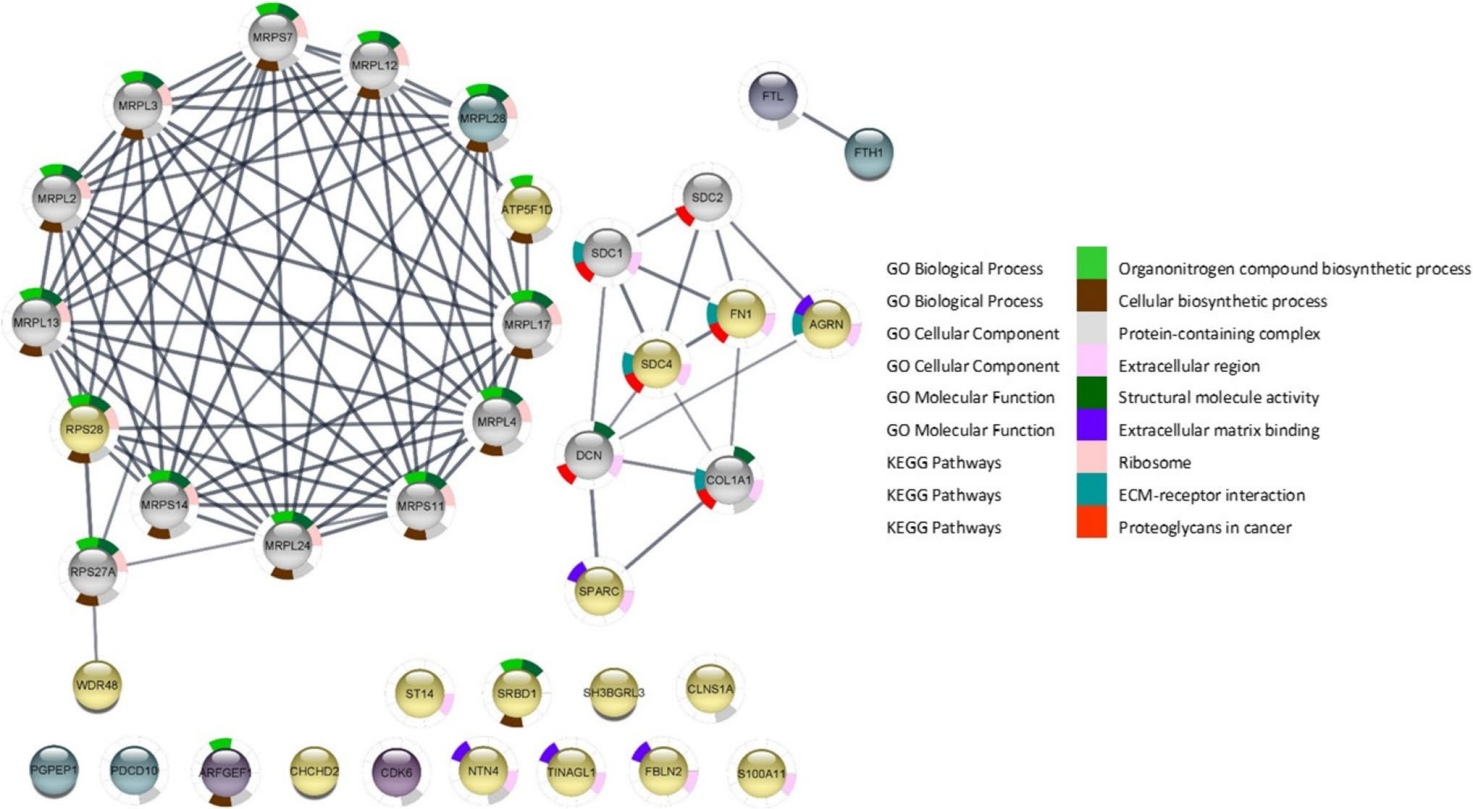
To determine the signalling pathways of the identified 40 DAPs: PPI network of 7.81 µM HFB_1_. The PPI network was constructed using string database and customised in Cytoscape and, where each node represents a protein, and edges represent the interaction between proteins. The PPI network consisted of 38 nodes and 85 edges. Downregulated DAPs in *Sus scrofa* intestinal cell line (IPEC-J2) are shown in yellow whereas the upregulated are shown in dark green-purple, other predicted proteins shown in grey. Interactions were predicted with a high confidence level of 0.700 and enrichment *p*-value < 0.05 was included in the analyses

In contrast, HFB_1_ exposure at 7.81 µM resulted in the downregulation of FN1 and revealed strong interactions with various syndecan and collagen pathways. Specifically, proteins such as syndecan-1 (SDC1), syndecan-2 (SDC2), syndecan-4 (SDC4), and collagen type 1 alpha 1 chain (COL1A1) were influenced by HFB_1_ exposure, which enriched the proteoglycans in cancer and ECM-receptor interaction pathways (Fig. 7). Syndecans are heparan sulphate proteoglycans involved in cellular adhesion, migration, and proliferation, and they are associated with colorectal cancer progression (Han et al. 2004; Park et al. 2002). In particular, SDC4 has been linked to ERK pathway activation, driving cellular proliferation (Chua et al. 2004; Corti et al. 2013). Collagen proteins, including COL1A1, are often mutated or altered during tumour progression to facilitate cellular invasion and metastasis (Xu et al. 2019). The disruption of collagen and syndecan pathways suggests that HFB_1_ exposure promotes cancer progression by modulating these pathways, particularly during cellular proliferation, migration, and adhesion processes. Although only a limited number of the identified DAPs were central within the PPI network, enrichment analysis revealed associations with cancer-related pathways such as proteoglycans in cancer and ECM-receptor interaction. Proteins like FN1 and COL1A1, despite representing a subset of the network, are known to play critical roles in cell adhesion, migration, and extracellular matrix remodelling, processes relevant to tumour progression. These findings suggest that even modest perturbations in key ECM-associated proteins may influence cancer-related signalling events.

**Fig. 8:**
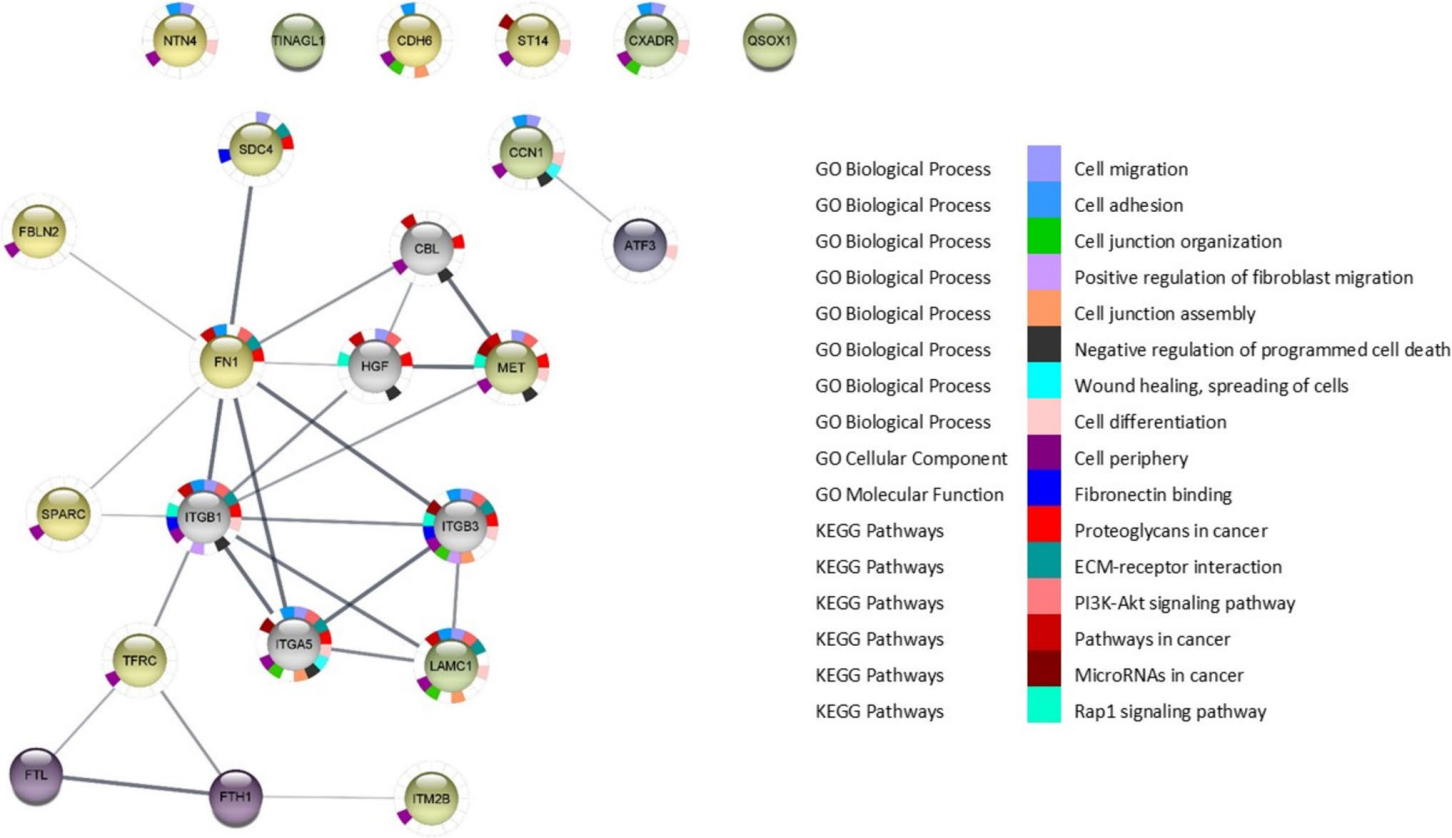
PPI network of 15.63 µM HFB_1_, to determine the signalling pathways of the 18 significant proteins identified; significant upregulated proteins in darker purple; whilst downregulated proteins shown in yellow. The PPI network consisted of 23 nodes and 26 edges

Unlike FB_1_, which primarily affects integrin pathways, HFB_1_ exposure engages alternative cancer-promoting mechanisms, potentially explaining the differential toxicity observed in vitro versus in vivo studies. The present study suggests that HFB_1_ toxicity may vary depending on its concentration and the specific pathways it influences. At the higher concentration (15.63 µM), HFB_1_ exposure led to a combination of integrin and syndecan pathway interactions, resulting in a broader range of cancer-promoting effects. Cytoscape analysis revealed interactions between FN1 and integrins (ITGA5, ITGB1), syndecans (SDC4), fibulin (FBLN2), and laminin subunit gamma 1 (LAMC1), all of which are involved in potent cancer-promoting pathways (Fig. 8). This enrichment pointed to the PI3K-Akt signalling pathway, wound healing, and additional cancer-related pathways, highlighting the complex and multifaceted impact of HFB_1_ at higher concentrations.

FBLN2, which is overexpressed in lung adenocarcinoma cell lines (Baird et al. 2013), interacts with the ECM through integrin, collagen, and laminin pathways to support cellular proliferation. The increased expression of LAMC1, associated with TGF-β signalling, has been linked to tumour progression and inflammatory microenvironments (Fang et al. 2021; Taniguchi and Karin 2018). These findings provide further evidence of the enhanced toxicity of HFB_1_ at higher concentrations, as it influences a broader array of cancer-related pathways compared to FB_1_.

## Conclusion

In summary, exposure to different concentrations of mycotoxins resulted in distinct protein interactions and enriched pathways, shedding light on the mechanistic differences between FB_1_ and HFB_1_ toxicity. The identification of FN1 regulation, along with the associated cancer-related pathways, offers potential mechanistic insights into the cellular responses to FB_1_ and HFB_1_. However, these findings cannot fully explain the differences reported between in vitro and in vivo outcomes, and further studies using more physiologically relevant models are needed to confirm these effects. The results from both the cell optimisation model and the proteomics analysis indicate that HFB_1_ exerts greater toxicity on the IPEC-J2 cell line, owing to the increased number of affected proteins and enriched pathways. This underscores the importance of considering mycotoxin concentration and the variety of molecular pathways involved when assessing their potential toxicity in intestinal cells. This study provides mechanistic insight into the cellular responses induced by FB_1_ and HFB_1_ exposure; however, the limitations of the in vitro model must be acknowledged. The absence of systemic interactions, metabolic activity, and immune modulation inherent to in vivo systems may influence the physiological relevance of the observed effects. Consequently, the findings should be interpreted within the context of the simplified cellular environment. Further in vivo investigations or advanced in vitro models (e.g. co-culture or organ-on-chip systems) are warranted to validate these results and better reflect the biological complexity of whole-organism exposure.

## Supplementary Information

Below is the link to the electronic supplementary material.

ESM1 (DOCX 453 KB)

## Data Availability

No datasets were generated or analysed during the current study.
