# Supplementary material for: Comparative proteomic analysis indicates differential responses to fumonisin B1 (FB1) and hydrolysed fumonisin B1 (HFB1) in IPEC-J2 porcine epithelial cells in vitro
Source: Mycotoxin Res. 2025 Oct 9;41(4):643–65. doi: 10.1007/s12550-025-00607-z (PMC12612002; doi:10.1007/s12550-025-00607-z)
Supplement: Supplementary file 1 — (DOCX 453 KB) [file 12550_2025_607_MOESM1_ESM.docx]

**Table S1.** Log₂ fold change and corresponding linear fold change values for differentially abundant proteins detected in the IPEC-J2 cells treated with 7.81 µM HFB_1_.

| **HFB_1_ (7.81 µM)** | | | |
| --- | --- | --- | --- |
| **Protein** | **log2FC** | **FC** | **adjusted p-value** |
| FN1 | -0,62785 | 0,64714 | 0,002809288 |
| FBLN2 | -0,80271 | 0,57327 | 0,00337131 |
| TINAGL1 | -0,48234 | 0,715814 | 0,003693208 |
| SDC4 | -0,66873 | 0,62906 | 0,005432047 |
| ST14 | -0,70569 | 0,61315 | 0,005529327 |
| FTL | 0,585054 | 1,500095 | 0,014805622 |
| RPS28 | -0,53565 | 0,689849 | 0,028120194 |
| FTH1 | 0,432449 | 1,349522 | 0,030758143 |
| SRBD1 | -0,56214 | 0,677295 | 0,031934043 |
| CLNS1A | -0,72142 | 0,606501 | 0,04449574 |
| WDR48 | -0,4651 | 0,724423 | 0,044828181 |
| ATP5F1D | -0,52626 | 0,694353 | 0,045170841 |
| PGPEP1 | 0,442219 | 1,358693 | 0,046011169 |
| PDCD10 | 0,429931 | 1,347169 | 0,046984787 |
| ARFGEF1 | 0,661006 | 1,581185 | 0,047058452 |
| NTN4 | -0,51799 | 0,698343 | 0,047577433 |
| SH3BGRL3 | -0,5938 | 0,662595 | 0,047976379 |
| MRPL28 | 0,425606 | 1,343137 | 0,049322393 |
| S100A11 | -0,54955 | 0,683232 | 0,049852607 |
| ui2 | -0,4192 | 0,74784 | 0,053816543 |
| SPARC | -0,72569 | 0,604707 | 0,054175801 |
| AGRN | -0,51591 | 0,69935 | 0,055166224 |
| CHCHD2 | -1,09352 | 0,468617 | 0,055225761 |
| CDK6 | 0,710724 | 1,636625 | 0,055944889 |
| CEP192 | 0,543931 | 1,45794 | 0,056748685 |
| RPS21 | -0,40724 | 0,754067 | 0,057028512 |
| CD44 | -0,44987 | 0,73211 | 0,057579244 |
| NFU1 | -0,42433 | 0,745185 | 0,05787728 |
| GCA | 0,453728 | 1,369574 | 0,058952414 |
| MRPL18 | 0,501435 | 1,415621 | 0,065786809 |
| CYB5B | -0,4103 | 0,752467 | 0,071012177 |
| ABRACL | 0,418783 | 1,3368 | 0,072146863 |
| CD276 | -0,73829 | 0,599448 | 0,073006871 |
| VEZT | 0,509592 | 1,423648 | 0,075154975 |
| TBC1D23 | 0,461704 | 1,377167 | 0,076895602 |
| CCN3 | -1,20758 | 0,432996 | 0,078490763 |
| ACTN2 | 0,614369 | 1,530888 | 0,081428541 |
| SKP1 | -0,435 | 0,739695 | 0,081635783 |
| GOLT1B | 0,438105 | 1,354823 | 0,0830065 |
| PPP1R2 | -0,63966 | 0,641864 | 0,087287575 |
| INTS3 | -0,64563 | 0,639214 | 0,093889105 |

**Table S2.** Log₂ fold change and corresponding linear fold change values for differentially abundant proteins detected in the IPEC-J2 cells treated with 15.63 µM HFB_1_.

| **HFB_1_ (15.63 µM)** | | | |
| --- | --- | --- | --- |
| **Protein** | **log2FC** | **FC** | **adjusted p-value** |
| TFRC | -0,67025 | 0,6284 | 3,2E-05 |
| FN1 | -0,77427 | 0,584686 | 0,000121 |
| QSOX1 | -0,46081 | 0,72658 | 0,000173 |
| FBLN2 | -0,86928 | 0,54742 | 0,000486 |
| SDC4 | -0,683 | 0,622867 | 0,000516 |
| NTN4 | -0,86606 | 0,548643 | 0,000716 |
| ATF3 | 0,452084 | 1,368015 | 0,001137 |
| FTL | 0,581029 | 1,495916 | 0,003149 |
| FTH1 | 0,618069 | 1,534819 | 0,003798 |
| ST14 | -0,77072 | 0,586124 | 0,004015 |
| CCN1 | -0,46843 | 0,72275 | 0,006193 |
| CXADR | -0,41445 | 0,750304 | 0,013045 |
| TINAGL1 | -0,43714 | 0,738598 | 0,031586 |
| LAMC1 | -0,41004 | 0,752604 | 0,031626 |
| ITM2B | -0,58653 | 0,665941 | 0,033122 |
| MET | -0,57431 | 0,671608 | 0,052956 |
| CDH6 | -1,1628 | 0,446646 | 0,070011 |
| SPARC | -0,79869 | 0,574871 | 0,092827 |

**Table S3.** Log₂ fold change and corresponding linear fold change values for differentially abundant proteins detected in the IPEC-J2 cells treated with 15.63 µM FB1.

| **FB_1_ (15.63 µM)** | | | |
| --- | --- | --- | --- |
| **Protein** | **log2FC** | **FC** | **adjusted p-value** |
| EHD2 | 0,416791698 | 1,334955543 | 0,006053688 |
| FN1 | 0,455386212 | 1,371149815 | 0,049063042 |
| CD276 | 0,555280119 | 1,46945392 | 0,052341275 |
| TGFBR2 | 0,46288782 | 1,378297976 | 0,052985518 |
| RBP4 | 0,771268351 | 1,706769637 | 0,05561925 |


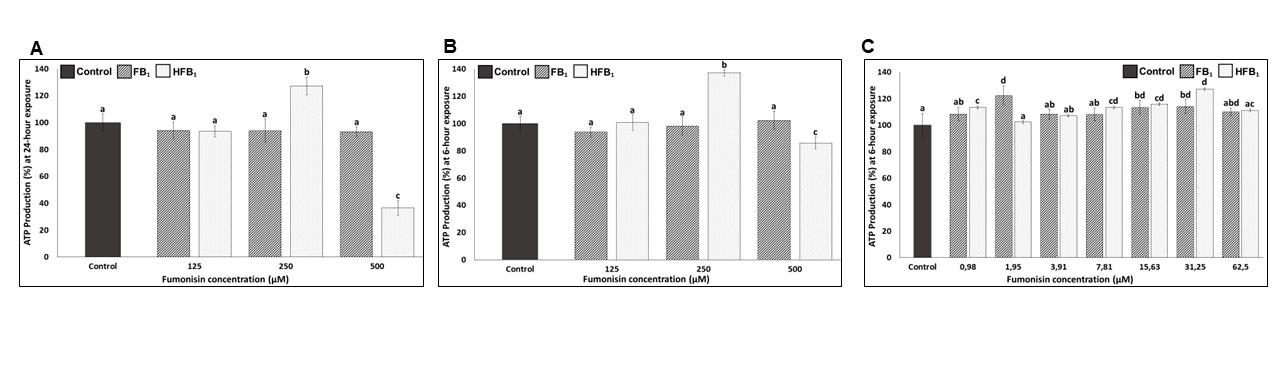


**Figure S1**. The effect of fumonisin B_1_ (FB_1_) and hydrolysed fumonisin B_1_ (HFB_1_) on cell viability (ATP production) at 6- (A, C) and 24-hour (B) incubation periods in IPEC-J2 porcine intestinal cells. ATP- adenosine triphosphate production was calculated as a percentage and at a statistical significance of p < 0.05. Concentrations include a control and various concentrations of FB1 and HFB1 (ranging from 0.98 µM to 500 µM). Statistically significant differences (p < 0.05) are shown as lowercase letters above each of the graph bars where each concentration of FB1 and HFB1 are compared to the control.


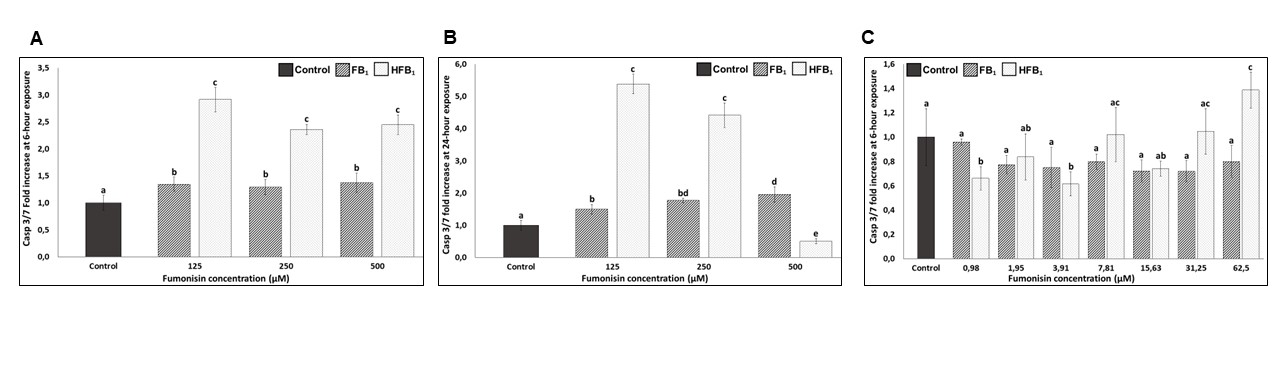


**Figure S2**. The effect of fumonisin B_1_ (FB_1_) and hydrolysed fumonisin B_1_ (HFB_1_) on apoptosis (Caspase 3/ 7-fold increase) at 6- (A, C) and 24-hour (B) incubation periods in IPEC-J2 porcine intestinal cells. Statistical analyses were included at a statistical significance of p < 0.05. Statistically significant differences (p < 0.05) are shown as lowercase letters above each of the graph bars where each concentration of FB_1_ and HFB_1_ are compared to the control.


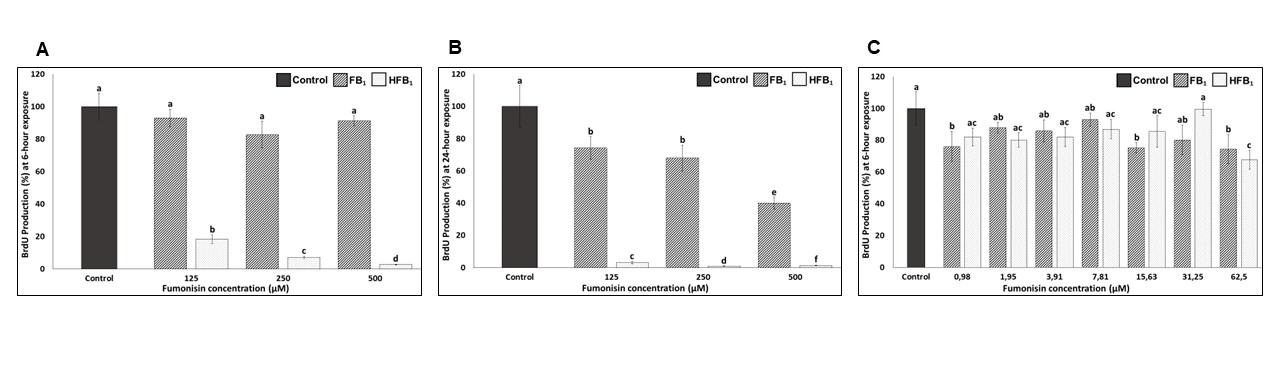


**Figure S3**. The effect of fumonisin B_1_ (FB_1_) and hydrolysed fumonisin B_1_ (HFB_1_) on cell proliferation (BrdU incorporation) at 6- (A, C) and 24-hour (B) incubation periods in IPEC-J2 porcine intestinal cells. BrdU- Bromodeoxyuridine was calculated and is shown as a percentage. Statistically significant differences (p < 0.05) are shown as lowercase letters above each of the graph bars where each concentration of FB_1_ and HFB_1_ are compared to the control.


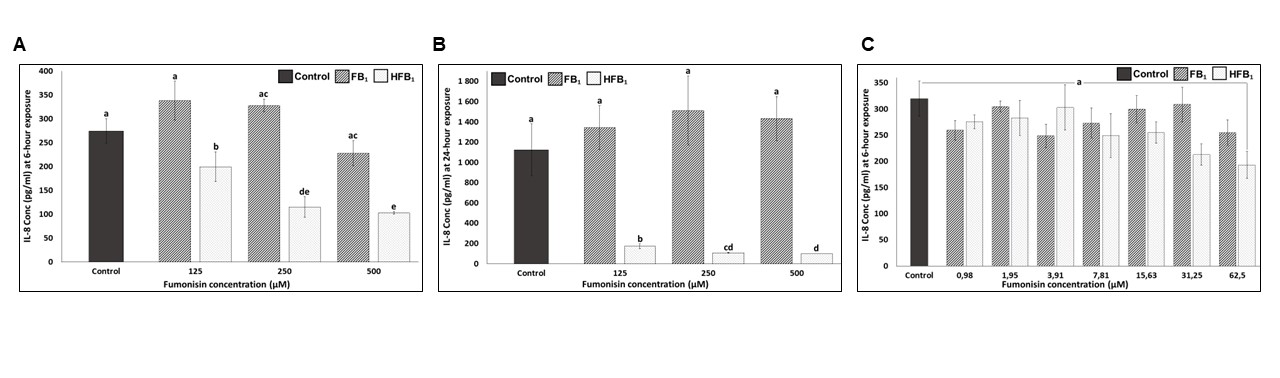


**Figure S4**. The effect of fumonisin B_1_ (FB_1_) and hydrolysed fumonisin B_1_ (HFB_1_) on an inflammatory biomarker (IL-8: interleukin 8 Conc: concentration) at 6- (A, C) and 24-hour (B) incubation periods in IPEC-J2 porcine intestinal cells. Statistically significant differences (p < 0.05) are shown as lowercase letters above each of the graph bars where each concentration of FB_1_ and HFB_1_ are compared to the control.

**
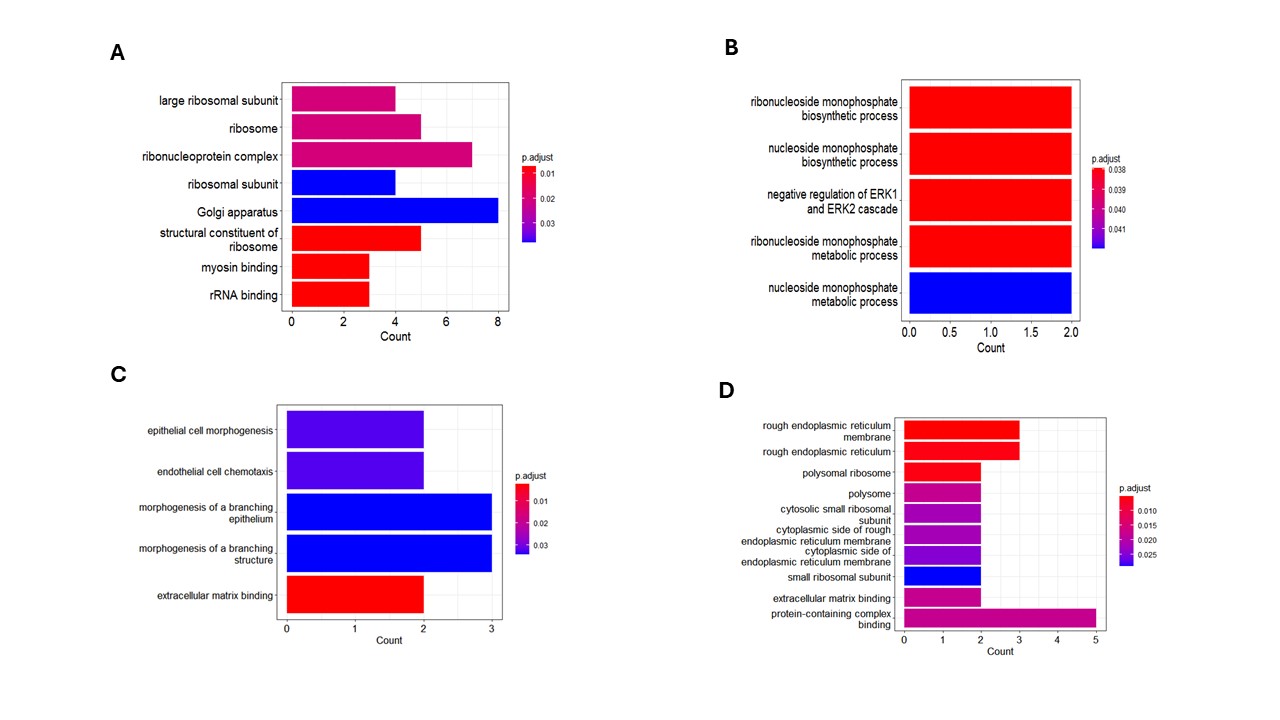
**

**Figure S5**. Gene Ontology overview (Biological Process, Cellular Components, and Molecular Function) of the significant down-regulated DAPs: A) HFB_1_ (7.81 µM) and B) HFB_1_ (15.63 µM) and up-regulated DAPs: C) HFB_1_ (7.81 µM) and D) HFB_1_ (15.63 µM) using the org.Ss.eg.db -Porcine database. All the GO terms are ranked by adjusted p-value. (Plots generated in cluster profiler in R).
